# Combined Use of Structure Analysis, Studies of Molecular Association in Solution, and Molecular Modelling to Understand the Different Propensities of Dihydroxybenzoic Acids to Form Solid Phases

**DOI:** 10.3390/pharmaceutics13050734

**Published:** 2021-05-16

**Authors:** Aija Trimdale, Anatoly Mishnev, Agris Bērziņš

**Affiliations:** 1 Faculty of Chemistry, University of Latvia, Jelgavas iela 1, LV-1004 Riga, Latvia; 2 Latvian Institute of Organic Synthesis, Aizkraukles iela 21, LV-1006 Riga, Latvia; mishnevs@osi.lv

**Keywords:** polymorphs, solvates, solvate formation, molecular association, crystal structure analysis, dihydroxybenzoic acid

## Abstract

The arrangement of hydroxyl groups in the benzene ring has a significant effect on the propensity of dihydroxybenzoic acids (diOHBAs) to form different solid phases when crystallized from solution. All six diOHBAs were categorized into distinctive groups according to the solid phases obtained when crystallized from selected solvents. A combined study using crystal structure and molecule electrostatic potential surface analysis, as well as an exploration of molecular association in solution using spectroscopic methods and molecular dynamics simulations were used to determine the possible mechanism of how the location of the phenolic hydroxyl groups affect the diversity of solid phases formed by the diOHBAs. The crystal structure analysis showed that classical carboxylic acid homodimers and ring-like hydrogen bond motifs consisting of six diOHBA molecules are prominently present in almost all analyzed crystal structures. Both experimental spectroscopic investigations and molecular dynamics simulations indicated that the extent of intramolecular bonding between carboxyl and hydroxyl groups in solution has the most significant impact on the solid phases formed by the diOHBAs. Additionally, the extent of hydrogen bonding with solvent molecules and the mean lifetime of solute–solvent associates formed by diOHBAs and 2-propanol were also investigated.

## 1. Introduction

When crystallized from a solution, at least one-third of all small organic compounds exhibit the ability to crystallize into multiple nonsolvated or solvated crystalline phases. Since the early 1990s, the phenomenon of polymorphism and solvatomorphism has been a subject of great interest [1]. The compounds’ ability to form multiple crystalline phases with differing thermodynamic parameters and physicochemical properties [2] makes molecular crystal engineering studies vitally necessary for many manufacturers including those in the pharmaceutical industry, as it is necessary to find the most suitable drug candidate with the desired properties and technologically the most appropriate and economically beneficial crystal phase for manufacturing. The interest from the industries and the rapid growth of computational power in this millennia led an ongoing development of multiple methods aimed to predict the formation of polymorphic and solvated phases and to provide insights into the fundamental trends related to the preparation and occurrence of crystalline phases [3].

The most widely used methods are crystal structure prediction (CSP) studies that provide a calculated crystal energy landscape of a compound and aim to determine the most stable crystal structures for either a single compound [4,5,6,7] or multicomponent solids [8,9]. CSP studies directly do not provide information about fundamental trends related to the formation of different crystalline phases. Another common group of studies employ the use of machine learning and data mining from the Cambridge Structural Database (CSD) to predict both polymorph and solvate formation propensity [10,11,12], and to distinguish fundamental trends related to the preparation and occurrence of solvates [13,14]. However, the fundamental question on why some molecules form numerous solvates while others form none still remains open [3,9,15].

Among the latest computational approaches, molecular modelling or molecular dynamics (MD) simulations can be highlighted [16]. Typical usage of MD revolves around molecular biology, association and folding of proteins [17], etc., whereas its employment for association studies of small molecules has gained popularity only quite recently. It has been used to investigate solvation, association [18], nucleation [19], processes and conformational changes [20] in solution, and also for the subject of interest of this article, i.e., solvate forming and nonforming compound molecule behaviour in solution [21].

Previous attempts to compare the solid form landscapes [9,22,23,24,25] or just crystal structures [26,27,28,29] of chemically highly similar compounds have led to conclusions that the crystal structures and solid form propensity in general is characteristic to each unique compound, although occasionally seeding or templating with chemically similar molecules can induce the formation of mutually isostructural forms [25,30,31]. It is reported that a high tendency to form a large number of solvates can be explained by a particular feature of the molecule preventing efficient packing in a pure form [23,24] or just by alternative energetically competative single-component and framework structures [9], but this is not the key factor in all cases [22]. 

In this study, we explore dihydroxybenzoic acids (diOHBAs) that are relatively well-characterized compounds of pharmaceutical relevance with multiple known solvated and nonsolvated phases [32,33,34,35,36,37,38,39] and, despite their structural similarity, have notably contrasting propensity to form different solid phases when crystallized from solution. Previous studies have utilized analysis of structural aspects in crystal forms of 2,3-, 2,5-, 3,4-, and 3,5-diOHBAs [38,39] and the CSP approach to predict hydrate formation for 2,4- and 2,5-diOHBAs [8] and to predict the polymorph formation for 2,4-diOHBA [34].

In our research, we focus on the determination of causes and the mechanism of how the location of the phenolic hydroxyl groups affects the diversity of solid phases formed by the diOHBAs, and in contrast to the previously published studies, we consider all six isomers of diOHBA. We employ a combined use of crystal structure analysis [23,40] and an analysis of molecule electrostatic potential surfaces [22,41,42], molecular behaviour, and association studies in solution using vibrational and NMR spectroscopy [43,44] and molecular dynamics simulations [45] to rationalize molecular interactions and hydrogen-bond formation propensity and to evaluate how the differences present as a result of a change of hydroxyl group position affect the diversity of solid phases formed by the diOHBAs.

## 2. Methods and Materials

### 2.1. Materials, Crystallization Experiments, and Crystal Form Identification

All six dihydroxybenzoic acids—2,3-diOHBA (>98%, form I), 2,4-diOHBA (>95%, form II), 2,5-diOHBA (>99%, form II), 2,6-diOHBA (>98%, form I and MH), 3,4-diOHBA (>95%, form I and MH II), 3,5-diOHBA (95%, form I)—were purchased from Fluorochem or Aldrich and used without further purification except for the mixtures with hydrates present, which prior to use were dehydrated for 24 h in 70/80 °C to obtain a pure polymorph. Organic solvents (acetonitrile (ACN), 1,4-dioxane (DXN), and tetrahydrofuran (THF), 2-propanol (IPA)) of analytical grade were purchased from commercial sources and used without further purification. For ^1^H-NMR and ^13^C-NMR studies, acetonitrile-d8 (99.8 atom % D) and 2-propanol-d7 (99.0 atom % D) were purchased from Eurisotop.

Evaporation crystallization experiments were performed by preparing saturated solutions (in water, acetonitrile, 1,4-dioxane, tetrahydrofuran, and 2-propanol) at room temperature; the obtained solutions were filtered and slowly evaporated at ambient conditions. For cooling crystallization, saturated solutions were prepared at elevated temperature (40–50) °C; the obtained solutions were filtered and crystallized in a closed vial at 5 °C. The obtained products were collected by filtration, air-dried, and analyzed by recording powder X-ray diffraction (PXRD) pattern.

PXRD patterns were measured at ambient temperature on a D8 Advance (Bruker AXS, Karlsruhe, Germany) diffractometer using Cu Kα radiation (1.54180 Å), equipped with a LynxEye position-sensitive detector. The tube voltage and current were set to 40 kV and 40 mA. The divergence slit was set at 0.6 mm, and the antiscatter slit was set at 8.0 mm. The diffraction patterns were recorded using a 0.2 s/0.02° scanning speed from 3° to 35° on 2θ scale.

Previously undescribed crystalline phases were additionally characterized by a thermal analysis (DSC/TG). Measurement for a 5–10 mg sample was performed on Mettler Toledo TGA/DSC 2 (Mettler Toledo, Greifensee, Switzerland) in open 100 µL aluminum sample pans and nitrogen flow (100 mL·min^−1^) by heating from 25 to 300 °C with a heating rate of 10 °C·min^−1^. 

Crystal structures of unreported crystalline phases were determined by single-crystal X-ray diffraction (SCXRD). Single-crystal intensities of 3,5-diOHBA S_ACN_, S_0.5 THF_, and 3,4-diOHBA S_0.5 DXN_ were collected on an XtaLAB Synergy-S Dualflex diffractometer (Rigaku Corporation, Tokyo, Japan) equipped with a HyPix6000 detector and microfocus sealed X-ray using Cu K_α_ radiation (λ = 1.54184 Å). A single crystal with approximate dimensions of 0.1 × 0.06 × 0.02 mm^3^ was fixed with oil in a nylon loop of a magnetic CryoCap and set on a goniometer head. The sample was cooled down to 150 K, and ω-scans were performed with a step size of 0.5°. Data collection and reduction were performed with the CrysAlisPro 1.171.40.35a software (Oxford Diffraction Ltd., Abingdon, UK). Structure solution and refinement were performed with SHELXT software that are parts of the CrysAlisPro suite and Olex2.refine.

### 2.2. Ab Initio Calculations

For calculations of electrostatic potential (ESP), initial molecular geometries were taken from structures deposited in the Cambridge Structural Database (CSD) (CACDAM [32], BESKAL01 [33], ZZZEEU08 [34], LEZJAB [35], LEZJAB01 [36], EDUWUW [37], WUYNUA [38] WUYPOW01 [38]).Structure optimization in the gas phase was carried out in Gaussian 09 [46] with the density functional theory (DFT) functional B97D and 6-31++G(d,p) basis set with the temperature and pressure fixed at the values of 298 K and 1 atm [47]. A natural bond orbital (NBO) analysis was executed by using M06-2X functional with aug-cc-pVDZ basis set. Further quantitative analysis of the molecular ESP surface and surface extrema were carried out in Multiwfn 3.7 [48], and a spacing of grid points of 0.15 Bohr was used. The obtained ESP surfaces and their extrema were visualized in VMD 1.9.3 [49].

Mercury 2020.2.0 was used for the crystal structure analysis of all known nonsolvated and hydrated phases as well as selected solvated diOHBA phases. Prior to the crystal structure analysis, structures of 2,5-, 2,6-, 3,4-diOHBA polymorph I and 3,4- and 3,5-diOHBA monohydrates were modified in Mercury to correct for disorder and to ensure the formation of proper hydrogen bond interactions.

The packing coefficients of crystal structures and hydrogen bond geometries were calculated by PLATON [50]. Pairwise intermolecular interaction energy calculations of crystal structures were performed in CrystalExplorer 17.5 at the B3LYP-D2/6-31G(d,p) level [51]. The sum over all pairwise interaction energies with molecules for which atoms are within 15 Å of the central molecule was used to estimate the lattice energy.

The calculations of vibrational frequencies of 2,6-diOHBA *anti* and *syn* conformers were performed in Gaussin 09 (Gaussian, Inc., Wallingford, CT, USA) [46]. Structure optimization was carried out in polarizable continuum model (PCM) modelling solvent media with the density functional theory (DFT) functional B97D and 6-31++G(d,p) basis set [47]. Vibrational frequencies were calculated using M06-2X functional with aug-cc-pVDZ basis set and the PCM solvation model. The calculated vibration frequencies were scaled by a factor of 0.947 [52].

### 2.3. Association Studies by Using FT-IR and NMR Spectroscopy

For FT-IR measurements, solutions with concentrations of 0.1 and 0.01 M in pure acetonitrile and 2-propanol were prepared. Additional spectra were recorded also in 1,4-dioxane, tetrahydrofuran and acetonitrile with a water additive (equimolar to the diOHBA). FT-IR spectra were recorded at ambient temperature (25 °C) by using a PerkinElmer Fourier FTIR spectrometer in a spectral region between 400 and 4000 cm^−1^ with a resolution of 4 cm^−1^. Attenuated total reflectance (ATR) mode by using diamond ATR crystal was used for solids; transmission mode using standard KBr cell (l = 100 μm) was used for solutions. Data from 10 to 16 scans were collected and combined, and further analysis was carried out by using PerkinElmer Spectrum 10.03.07. software (PerkinElmer, Waltham, MA, USA).

For NMR measurements, 2,3-, 2,6-, and 3,5-diOHBA solutions with concentrations 10, 25, 50, 100 and, if solubility allowed, 200 mM in acetonitrile-d8 and 2-propanol-d7 were prepared. NMR spectra were recorded at ambient temperature by using Bruker Fourier 300 MHz. The number of scans was selected to obtain an acceptable signal-to-noise ratio (8–32 for ^1^H and 256–40,960 for ^13^C spectra). NMR spectra were processed using MestReNova 12.0 (Mestrelab Research, Santiago de Compostela, Spain). ^1^H NMR signals were referenced against TMS, and ^13^C spectra were referenced against solvent signal (1.39 ppm for acetonitrile CH_3_ and 25.8 ppm for 2-propanol CH_3_). Chemical shifts were allocated by using chemical shift values predicted using the MestReNova 12.0 software. All spectra of the solutions were recorded shortly after their preparation.

### 2.4. Molecular Dynamics (MD) Simulations

Unbiased molecular dynamics (MD) simulations were performed to investigate molecule behavior in solution using an explicit representation of the solvent. Molecular geometry and topology files for 2,3-, 2,4-, 2,6- 3,4-, and 3,5-diOHBA were generated with the General Amber Force Field (GAFF) using the standard GAFF procedure [53] with AmberTools19 [54] using molecular geometry directly taken from crystal structures (CACDAM [32], ZZZEEU08 [34], LEZJAB [35], LEZJAB01 [36], WUYNUA [38], WUYPOW01 [38]). Molecular geometry and topology files for solvents (acetonitrile, tetrahydrofuran, 2-propanol) were taken from the Virtual Chemistry database [55,56]. The initial configuration used in MD simulations was prepared by randomly inserting 24 diOHBA molecules in a cubic box (a = 6.5 nm) and then filling the box with solvent molecules resulting in a final concentration of ≈0.1–0.15 M. MD simulations were carried out using Gromacs 2019.2 [57]. The initial configuration was initially energy minimized with a steepest descent algorithm imposing an upper limit on the residual force of 1000 kJ mol^–1^·nm^−1^ and then equilibrated in the canonical (NVT) and isothermal–isobaric (NPT) ensembles for 100 ps. For each simulation, a time step of 2 fs was used. The production runs were carried out in the NPT ensemble for 100 ns at a pressure of 1.0 bar and a temperature of 300 K, using the Parrinello–Rahman barostat [58] and the Bussi–Donadio–Parrinello thermostat [59]. System coordinates were saved every 10 ps for further analysis.

The analysis of the distribution of intermolecular diOHBA distances and their relative orientations was performed using PLUMED 2 [60,61]. The solute’s center of mass and the vector connecting atoms C4 to C7 were used to define the position and absolute orientation of the solute molecules. An in-house python script [45] was used for collecting and plotting the data. The analysis of probability density distribution of hydrogen-bonded dimers and hydrogen-bonded associates with solvent molecules was performed using the VMD [49] HBonds plugin by counting the number of hydrogen bonds between the respective oxygen atoms that have a geometry corresponding to a conventional hydrogen bond (the distance between the donor (D) and acceptor (A) is less than 3.0 Å, and the angle D-H-A is 180 ± 20°). The solvent molecule escape time distribution for solute–solvent associates was obtained by postprocessing the atomic trajectories with PLUMED 2 [60,61] using a distance-only criterion for the identification of the bonded state, with a cut-off distance of 3 Å. The mean lifetime of the solute–solvent associates was obtained by fitting the solvent molecule escape time distribution to an exponentially decaying function.

## 3. Results and Discussion

### 3.1. Crystal Form Screening and Crystal Structure Evaluation

**Figure 1:**
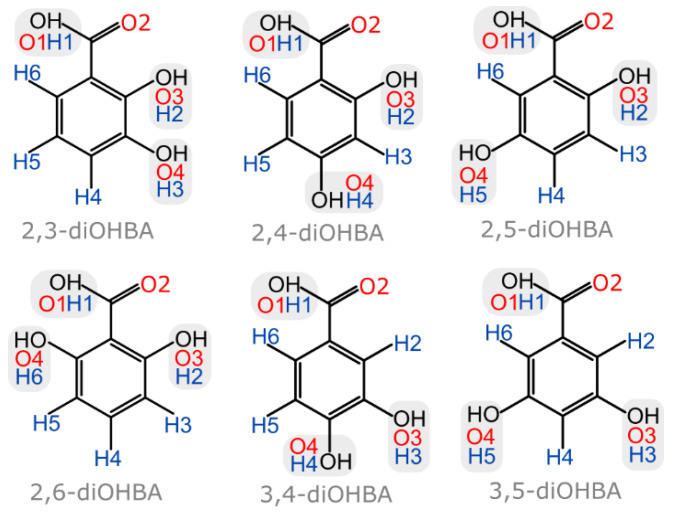
Structures of diOHBA with numbering of oxygen and hydrogen atoms.

To compare the solid form landscape of diOHBAs (see Figure 1), we performed crystallization of these compounds from 20 common solvents, which showed distinctive tendencies in solid form formation propensity among all the six isomers of diOHBA. For phase identification, the powder X-ray diffraction (PXDR) patterns of all the obtained products were compared to those simulated from crystal structures deposited in the CSD (see Figure S17 in the Supplementary Materials). In these experiments, multiple obtained crystalline phases could not be identified as polymorphic form or solvates deposited in the CSD and thus were additionally characterized. All the pure previously unreported crystalline phases were also thus analyzed using a thermal analysis (DSC/TG) to understand the solvent content and phase transformations of these phases. The obtained DSC/TG traces can be found in Figures S1–S6 in the Supplementary Materials. Crystal structure determination of the previously unreported 3,4- and 3,5-diOHBA phases was attempted using SCXRD.

In this article we focus on the results obtained by the crystallization and slow evaporation from acetonitrile (ACN), 1,4-dioxane (DXN), tetrahydrofuran (THF), 2-propanol (IPA), and water, and these data were also complemented by spectroscopic studies and MD simulations of the respective solutions. A summary of the crystal forms obtained from the above-highlighted solvents is given in Table 1, while data from the rest of the solvents can be found in Table S1 in the Supplementary Materials. Crystallographic information of the previously uncharacterized solvates (3,4-diOHBA 1,4-dioxane hemisolvate, 3,5-diOHBA acetonitrile solvate, and tetrahydrofuran hemisolvate) can be found in Table S2 in the Supplementary Materials. The structure of 3,5-diOHBA monohydrate could not be determined due to disorder problems as already previously noted by Sarma et al. [38].

From Table 1 we can clearly see that the small structural differences of these molecules, i.e., the arrangement of hydroxyl groups in the benzene ring, have a significant effect on the propensity to form different solid phases. Based on the observed tendencies in the propensity to form solid phases in the chosen solvents, we grouped the compounds into four distinctive groups: Group A (in the selected solvent only one polymorphic form was obtained), Group B (two polymorphs can be obtained, but mostly the most stable one was obtained), Group C (prone to form hydrate but no solvate in the given solvents was obtained), and Group D (extensively forms hydrates and solvates, as nonsolvated phases are complicated to obtain in crystallization). 

**Figure 2:**
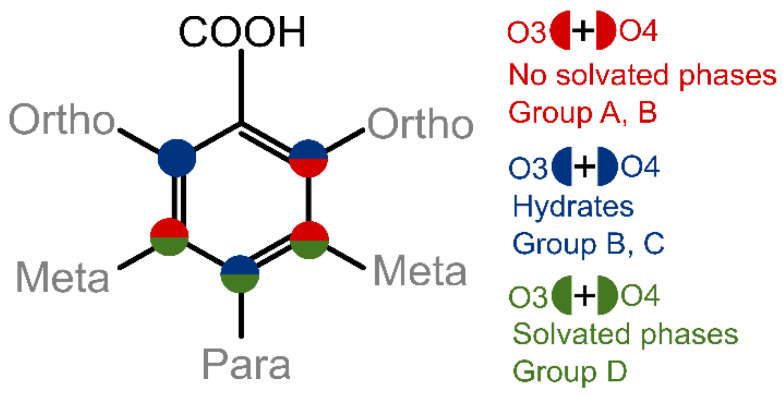
Schematic representation relation between the position of both phenolic hydroxyl groups and the overall propensity of diOHBAs to form solvated solid phases. Compound grouping as used in Table 1 is also given.

The most easily noticeable link between the structure of diOHBAs and solvate formation propensity is the position of hydroxyl groups: if the molecule has one of the phenolic hydroxyl groups in *ortho* position and the other one in *meta* position (2,3- and 2,5-diOHBAs), it does not show the propensity to form any solvates, while if the other hydroxyl group is not in the *meta* position (2,4- and 2,6-diOHBAs), hydrates are easily formed. In contrast, if there is no hydroxyl group in *ortho* position (3,4- and 3,5-diOHBAs) thus precluding the formation of an intramolecular hydrogen bond between the phenolic hydroxyl groups and carboxyl group, the compound readily forms multiple solvated forms. A schematic representation of this relation between the position of the phenolic hydroxyl groups’ overall propensity to form solvated solid phases and the grouping of the compounds as used in Table 1 is given in Figure 2.

Insight into the different propensities of diOHBAs to form solvated phases was also obtained by analyzing the hydrogen bond motifs in crystal structures of nonsolvated, hydrated, and solvated forms:

- 2,3-diOHBA: polymorph I (CACDAM [32]), polymorph II (CACDAM01 [38]);
- 2,5-diOHBA: polymorph I (BESKAL01 [33]), polymorph II (BESKAL08 [62]);
- 2,4-diOHBA: polymorph I (ZZZEEU08 [34]), polymorph II (ZZZEEU04 [63]), hemihydrate (QIVTUK [26]), monohydrate (YUXGUAV [64]);
- 2,6-diOHBA: polymorph I (LEZJAB01 [36]), polymorph II (LEZJAB [35]), monohydrate (LEZJEF [35]);
- 3,4-diOHBA: polymorph I (WUYNUA [38]), monohydrate I (BIJDON03 [26]), monohydrate II (BIJDON04 [38]), acetonitrile solvate (EDUWUW [37]), 1,4-dioxane hemisolvate;
- 3,5-diOHBA: polymorph I (WUYPOW01 [38]), polymorph II (WUYPOW [38]), hemihydrate (OKEMAT [39]), tetrahydrofuran solvate monohydrate (WUYPIQ [38]), tetrahydrofuran hemisolvate, acetonitrile solvate, 1,4-dioxane solvate (WUYPEM [38]).

Characteristic hydrogen bond motifs in crystal structures of all known nonsolvated phases of all the diOHBAs and the corresponding hydrogen bond geometric parameters, as well as characteristic hydrogen bond motifs in crystal structures of selected hydrated and solvated forms, can be found in Tables S4–S10 and Figures S10–S16 in the Supplementary Materials.

**Figure 3:**
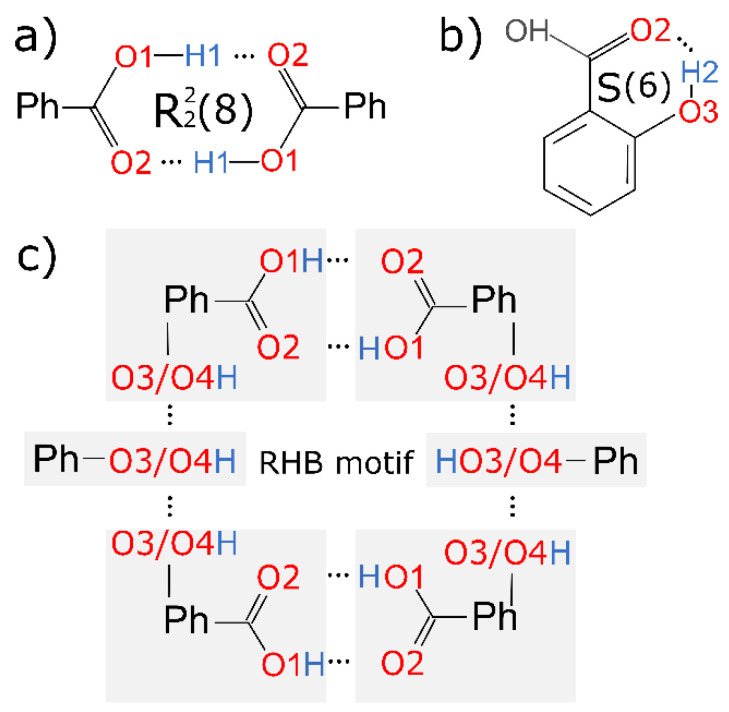
Schematic representation of the most characteristic hydrogen bond motifs in diOHBA crystal structures: (**a**) carboxyl acid homodimers R^2^_2_(8) formed by O1–H1^…^O2, (**b**) S(6) motif formed by intramolecular hydrogen bond O3–H2^…^O2, (**c**) ring-like hydrogen bond motif (RHB motif) consisting of two carboxyl acid homodimers R^2^_2_(8) interconnected by 2 additional diOHBA molecules via interactions between the phenolic hydroxyl groups.

All nonsolvated and hydrated structures of diOHBAs that have the hydroxyl group in *ortho* position contained an intramolecular hydrogen bond S(6) formed by O3–H2^…^O2 as the main hydrogen bond motif (see the schematic representation in Figure 3b). An almost equally common motif for all diOHBAs was carboxyl acid homodimer R^2^_2_(8) formed by O1–H1^…^O2, absent only in 2,6-diOHBA MH, polymorph II, 3,5-diOHBA polymorph II and 3,4-diOHBA hemihydrate (see the schematic representation in Figure 3a).

Nonsolvated phases containing the carboxylic acid homodimer R^2^_2_(8) nearly always also contained ring-like hydrogen bond motifs formed by six diOHBA molecules—R^12^_14_(34) in 2,3-diOHBA polymorph I, R^6^_8_(36) in polymorphs of 2,4-and 2,5-diOHBA, R^6^_8_(36) in 3,4-diOHBA polymorph I, and R^6^_6_(40) in 3,5-diOHBA polymorph I (see Table S3–S8 and Figures S10–S16 in the Supplementary Materials). This ring-like hydrogen bond motif (further abbreviated as RHB motif) consists of two carboxyl acid homodimers R^2^_2_(8) interconnected by 2 additional diOHBA molecules via interactions between the phenolic hydroxyl groups. A schematic representation of the RHB motif can be seen in Figure 3c. We also noted an interesting observation that the nonsolvated forms where such a six-membered ring motif is absent can be obtained only in crystallization from few specific solvents (2,3-diOHBA polymorph II) or in specific conditions (2,6-diOHBA polymorph I) or cannot be obtained in crystallization from solutions (3,5-diOHBA polymorph II). The RHB motif is also present in solvates of Group D compounds—in 3,5-diOHBA solvated phases (all except for the S_DXN_, probably because of the ability of 1,4-dioxane to form two hydrogen bonds) and 3,4-diOHBA acetonitrile solvate(the solvent molecules are located in channels enclosed by diOHBA molecules forming the RHB motif). This indicates that this motif is an essential construct not only for the formation of most of the nonsolvate phases, but also for the formation of solvated forms, in case the RHB motif is spacious enough to accommodate numerous different guest molecules.

**Figure 4:**
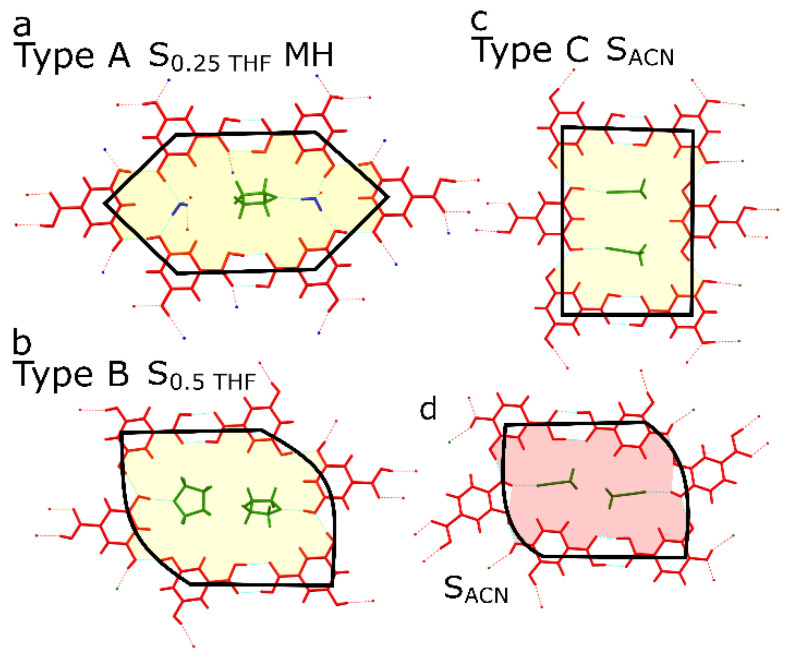
(**a**–**c**) The identified types of 3,5-diOHBA solvated forms based on the RHB motif in their structure. Type A solvate: the most common 3,5-diOHBA solvate type; RHB motif has an elongated shape, and water molecules are mandatory. Type B solvate: RHB motif is skewed, and water molecules are not mandatory. Type C solvate: rectangular RHB motif is identical to the RHB motif in 3,5-diOHBA polymorph I. (**d**) RHB motif in 3,4-diOHBA acetonitrile solvate.

The number of solvated phases formed by the 3,5-diOHBA makes this compound unique among all the diOHBAs. Interestingly, the structure of these solvates are not diverse and is in fact similar to that of the nonsolvated polymorph I. Previously, Varughese et al. [39] proposed a mechanism for the thermal transformations of 3,5-diOHBA solvates: after the loss of guest and water molecules, the RHB motifs that are present in solvates transform to the rectangular RHB motif of polymorph I. We further propose a classification of 3,5-diOHBA solvates into three distinctive types depending on the RHB motifs present in solvates. The most common solvates are Type A solvates in which there is an elongated RHB motif (Figure 4a). Water molecules are mandatory to stabilize this structure via O_water_–H_water_^…^O4/O3 and O3/O4H^…^O_water_ hydrogen bond and also bond solvate molecules to the 3,5-diOHBA (O_solvate_^…^H_water_O). There are two solvates classified as Type B solvates, i.e., S_0.7DMSO_HH and the previously undescribed S_0.5THF_, which0 could be obtained only when directly crystallized from THF in the absence of moisture. Type B solvates have a skewed RHB motif (Figure 4b), and the presence of water molecules is not mandatory. Furthermore, a RHB motif resembling that in Type B solvates is also found in 3,4-diOHBA acetonitrile solvate (Figure 4d). The previously undescribed S_ACN_ contains an identical RHB motif to that in 3,5-diOHBA polymorph I and is classified as Type C solvate (Figure 4c). This solvate does not contain water molecules in the structure. However, we highlight that this solvate is extremely unstable if compared to other rather stable 3,5-diOHBA solvates, which could indicate that the RHB motif present in this structure is not particularly stable.

We also noticed that alongside the RHB motifs phenolic hydroxyl groups form infinite hydrogen bonded chains in the structures of 2,5-diOHBA (Group B compound) polymorphs I (C(2)) and II (C^2^_2_(4)) and 3,5-diOHBA polymorph I (C(7)). However, only in 2,5-diOHBA are these chains an essential part of the R^6^_8_(36) RHB motif interconnecting the homodimer pairs and thus additionally stabilizing the structures which can be associated with no detected propensity to form structures with guest molecules in the structure. Instead, the R^6^_6_(40) RHB motif in 3,5-diOHBA encloses two parallel C(7) chains (O4^…^H3–O3) identically as the solvent molecules are enclosed in the Type C solvate, and these interconnect layers consist of RHB motifs (O1–H^…^O3_B_; O2^…^H–O1_B_; O3^…^H–O3_A_; O4^…^H–O4_A_, where indexes represent the identity of symmetrically unique molecules) in the same way as the guest molecules in the Type C solvate (formation of such complex motifs is allowed by 3 molecules in the asymmetric unit).

The proximity of the carboxyl group and hydroxyl groups in Group A and C compounds 2,3- and 2,6-diOHBAs results in the inevitable formation of multiple intermolecular hydrogen bonds (which also notably affect the electronic properties of both hydroxyl groups, see below) resulting in these compounds being different from the Group B compounds 2,4- and 2,5-diOHBAs. As a result, 2,3-diOHBA (Group A compound) forms a rather unique motif R^3^_3_(8) which, among other structures, is present only in 3,5-diOHBA polymorph II (reported to be obtained from the melt [38]), formed by an odd number (three) of molecules (O4_1_–H3_1_^…^O2_2_–C1_2_–O1_2_–H1_2_^…^O4_3_–H3_3_^…^O4_1_ where indexes represent the identity of molecules in the motif), and a RHB motif R^12^_14_(34) that connects two R^2^_2_(8) and two R^3^_3_(8) motifs (formation of such different motifs is allowed by 2 molecules in the asymmetric unit). Additionally, in the 2,3-diOHBA polymorph II (reported to be obtained by sublimation [38]), homodimers are linked directly by O4–H3^…^O1 without any additional linker molecule forming four-membered R^6^_6_(12), and the absence of the usual RHB motif could explain why the formation of this form can be obtained only from specific solvents.

**Figure 5:**
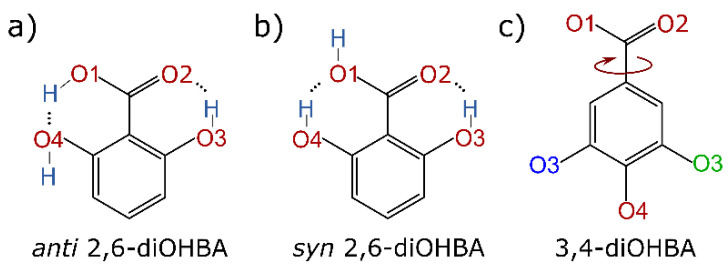
Representation of different conformation of 2,6-diOHBA and 3,4-diOHBA present in the crystal structures, for 2,6-diOHBA also showing intramolecular hydrogen bonds present. (**a**) *Anti* conformation of 2,6-diOHBA in polymorph II; (**b**) *Syn* conformation of 2,6-diOHBA in polymorph I; (**c**) conformation differences for 3,4-diOHBA associated with the relative arrangement of the carboxyl group and the O3 atom. Green—location of O3 atom as in all other diOHBAs. Blue—location of O3 atom as in solvates and polymorph I.

The 2,6-diOHBA (Group C compound) is in several ways unique among all six diOHBAs. Firstly, an *anti*-conformer (see Figure 5a) can be found in the crystal structures of polymorph II and monohydrate. Secondly, in 2,6-diOHBA polymorphs I and II, intramolecular hydrogen bonds S(6) (O3–H2^…^O2 (both polymorphs), O1–H1^…^O4 (polymorph II), and O4–H6^…^O1 (polymorph I)) (see Figure 5a,b) are formed by both phenolic hydroxyl groups and the carboxyl group by thus heavily affecting the molecule’s ability to form intermolecular hydrogen bonds. Therefore, in polymorph II, the only intermolecular hydrogen bond motif is chains C^2^_2_(6). The formation of homodimers R^2^_2_(8), as in polymorph I (reported to be obtained from hot toluene [36]), do not allow for the formation of any other intermolecular hydrogen bond. The introduction of water molecules, however, increases the diversity of hydrogen bond motifs present in the structure.

**Figure 6:**
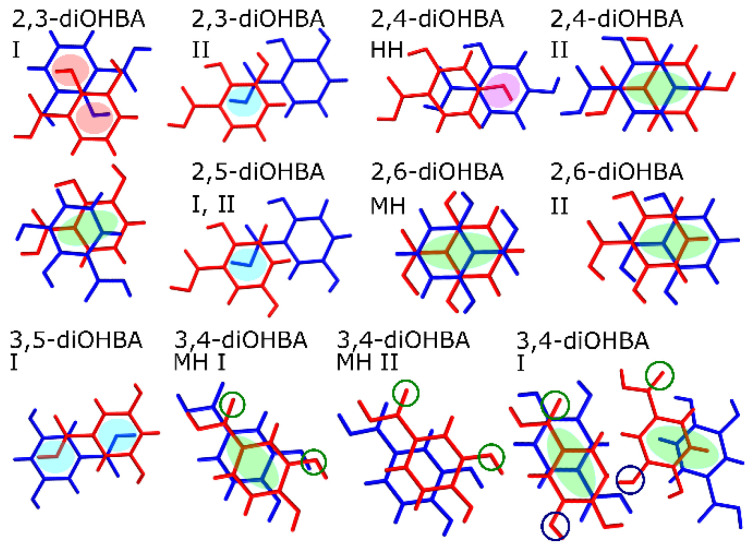
π–π interactions in crystal structures of diOHBA. Blue highlights—interaction between the benzene ring and O1 carboxylic hydroxyl groups; red and pink highlights—interaction between benzene ring and phenolic hydroxyl groups; green highlights— shifted π^…^π stacked molecules Encircled atoms highlight the conformation differences in 3,4-diOHBA due to the change in the relative arrangement of the carboxyl group and benzene ring.

In addition to hydrogen bond motifs, we also noticed conformation differences in the relative arrangement of the carboxyl group in the crystal structures of 3,4-diOHBA. In the crystal structures of all other diOHBAs, the *ortho*- or *meta*-positioned phenolic hydroxyl group O3 was always located next to the O2 atom. However, for 3,4-diOHBA this conformation was present only in the crystal structures of both hydrates, while in acetonitrile and 1,4-dioxane solvates and in polymorph I rotation of the carboxyl group resulted in conformation in which the O3 phenolic hydroxyl group is located next to the O1 atom (for visual representation of conformation differences see Figure 5c and encircled 3,4-diOHBA O atoms in Figure 6). These conformation differences between 3,4-diOHBA and other diOHBAs could be related to the fact that 3,4-diOHBA is more prone to form hydrates instead of solvates or polymorph I, given that in the hydrate conformation identical to that in other diOHBA is present.

A common structure feature in nonsolvated and hydrated phases is the presence of layers formed by R^2^_2_(8) bonded dimers or even R^2^_2_(8) dimer formed RHB motifs (this is also typical for the majority of 3,5-diOHBA solvates, as previously described by Varughese et al., who categorized solvated phases into four classes based on the topology, mode, and the extent of the empty space created [39]) that are interconnected by π^…^π interactions. Only the 2,4-diOHBA polymorph I does not form such a layered structure. However, in none of the structures were classically stacked π^…^π molecules observed. Instead, Group A and B compounds tend to stack in a way that leads to an interaction between the benzene ring and carboxylic hydroxyl groups O1 atom (2,3-diOHBA polymorph II and both 2,5-diOHBA polymorphs, with a distance of 3.30 Å between molecule planes; see blue highlights in Figure 6), and between the benzene ring and phenolic hydroxyl groups (2,3-diOHBA polymorph I and 2,4-diOHBA hemihydrate, the distance between molecule planes averaging at 3.3 Å; see red and pink highlights in Figure 6). Although MH and polymorph II formed by Group C compound 2,6-diOHBA do not contain the R^2^_2_(8) motif, in these structures and also in 2,4-diOHBA polymorph II (Group A) and 2,3-diOHBA polymorph I (Group B) there are shifted π^…^π stacked molecules (distance between molecule planes range 3.3–3.4 Å; see Figure 6 green highlights).

In addition, crystal structures of group D compounds contain both interactions between the benzene ring and the carboxylic hydroxyl groups O1 atom (3,5-diOHBA polymorph, the distance between molecule planes being 3.30 Å) and shifted π^…^π stacked molecules (3,4-diOHBA polymorph I and MH I, distance between molecule planes being 3.3–3.5 Å). These interactions are typical also for the majority of Group D compound solvated phases. However, not all solvated or hydrated structures contain π^…^π interactions (for example, the distance between molecule planes in 3,4-diOHBA MH II is 5.2 Å).

An additional crystal structure characterization of the nonsolvated forms was performed by calculating the packing index and lattice energy (see Table S3 in the Supplementary Materials). Although it has been identified that one of the potential driving forces resulting in facile solvate formation is the inefficient packing of nonsolvated phases [23,65], the studied benzoic acid derivatives are small molecules able to pack rather efficiently, and the observed rather low packing index differences (values ranging from 70.6 to 74.1% for the experimentally obtained forms) do not affect the solvate formation, similarly as for previously analyzed similarly sized molecules [22]. Likewise, the lattice energy of nonsolvated forms also was not able to provide information on the propensity to form solvates, and the calculated values for nearly all structures instead correlate with the ability of the compound to form intermolecular hydrogen bonds (in general, the lower the number of intramolecular hydrogen bonds, the lower the lattice energy).

### 3.2. Analysis of the Electrostatic Potential of diOHBA

**Figure 7:**
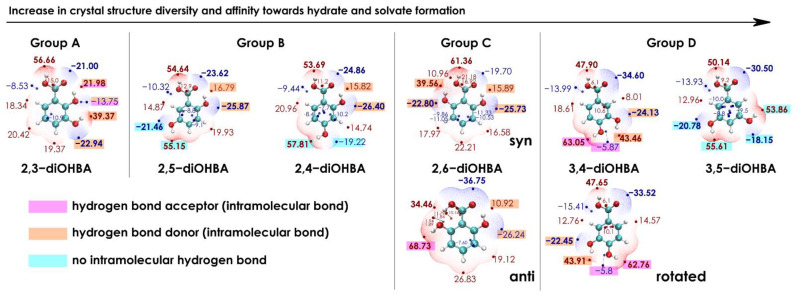
Electrostatic potential (ESP) surfaces and ESP extrema of all six diOHBAs arranged based on the overall structure diversity of the compounds and the number of hydrates and solvates obtained, by showing *syn* and *anti* conformers of 2,6-diOHBA and both conformers of 3,4-diOHBA associated with the relative arrangement of the carboxyl group. Values of ESP extrema are given in kcal·mol^−1^. For each compound, numerical values of ESP extrema associated with hydroxyl groups and carboxyl group are shown; those of considerable magnitude (greater than values associated with the phenolic hydrogens) are highlighted in bold, while values associated with hydroxyl groups are colored according to the involvement of the hydroxyl group in intramolecular hydrogen bonds.

The difference in the ability of diOHBAs to form multiple crystal forms could be associated with the differences in propensity to form different intermolecular interactions. One of the tools that could be used for exploring this feature is ESP surfaces [66,67]. ESP surfaces generated for diOHBAs are given in Figure 7 and are arranged from left to right according to the grouping as in Table 1 (the observed tendencies in the propensity to form solid phases in the selected solvents). Based on the above-given analysis of the known crystal structures, carboxylic acid dimers are present in most structures, and interactions with solvent molecules are mostly formed by the phenolic hydroxyl groups; thus, to rationalize the affinity of diOHBAs to form solvated forms, ESP surfaces of isolated diOHBA molecules were generated.

By considering only the numerical values, 2,3-diOHBA (Group A compound) is the only molecule for which the value of ESP extremum located on a non-*ortho*-hydroxyl group (39.37 kcal·mol^−1^) is notably lower than that located on the H1 (56.66 kcal·mol^−1^). This is also the only molecule in which a phenolic hydroxyl group is involved in the formation of two intramolecular hydrogen bonds and acts both as the donor and acceptor, thus hindering the ability of this molecule to bond with other molecules.

For 2,5-diOHBA and 2,4-diOHBA (Group B compounds), the numeric values of the ESP surface extrema on the phenolic and carboxylic hydroxyl groups are similar. Both compounds have a free hydroxyl group (O4) that can interact with other molecules and can act as a good hydrogen donor and/or acceptor. This is utilized, for example, by the formation of 2,4-diOHBA hemihydrate.

For 2,6-diOHBA (Group C compound), both conformations found in crystal structures were considered. The *syn*-conformer, for which both phenolic hydroxyl groups are involved in intramolecular hydrogen bonds as hydrogen bond donors, is the only case when the negative extremum located on the carboxyl group (−19.70 kcal·mol^−1^) has a lower absolute value than for those on the phenolic hydroxyl groups (−22.80, −25.73 kcal·mol^−1^). The phenolic hydroxyl groups of the more common *anti*-conformer are also involved in intramolecular hydrogen bond formation; however, H6 can still easily interact with other molecules. Furthermore, the extremum on H6 (68.73 kcal·mol^−1^) has the largest positive value among all six diOHBAs, indicating that H6 is potentially a very good hydrogen bond donor. This is confirmed by all three known structures in which H6 acts as a hydrogen bond donor. The *anti*-conformer also has similarities to the other pronounced hydrate former 3,4-diOHBA: the numeric values of the ESP extrema on the phenolic hydroxyl groups for both compounds are similar.

By summarizing the observations for all *ortho*-substituted diOHBAs, the positive extrema of the ESP surface are not evenly distributed across the molecule since at least one of the hydroxyl groups forms an intramolecular hydrogen bond with the carboxyl group. Additionally, if none of the phenolic hydroxyl groups are free to act as a hydrogen bond donor when interacting with other molecules, the formation of hydrates and solvates is unlikely, since based on the known structures of the solvated forms of diOHBAs, solvent molecules mostly bond with the phenolic hydroxyl groups.

Unlike for *ortho*-substituted diOHBAs, for both 3,4- and 3,5-diOHBAs (Group D compounds), the ESP extrema values on the phenolic hydroxy groups do not significantly differ from each other and from the extremum on the carboxylic group, and all three positive ESP extrema are evenly distributed across the molecule. The relative arrangement of the carboxyl group with respect to the benzene ring in 3,4-diOHBA has a negligible effect on the ESP extrema values. For 3,4-diOHBA, the most positive extremum is located on H4 (63 kcal·mol^−1^), while all three positive extrema of 3,5-diOHBA have similar values (50–56 kcal·mol^−1^). In addition, 3,5-diOHBA is the only compound for which both phenolic hydroxyl groups are not involved in any intramolecular hydrogen bond and can freely interact with solvent molecules. Additionally, for both compounds, the ESP surface extrema on the carboxyl group have lower values than for other diOHBAs (except for the *anti*-conformer of 2,6-diOHBA), indicating a different affinity towards the formation of intermolecular interactions involving the carboxylic group, which could affect the tendency to form carboxylic acid dimers.

### 3.3. FTIR, ^1^H, ^13^C NMR Spectroscopy Studies of Association of diOHBAs in Solutions

One of the ways to try to rationalize the propensity of a compound to form different solid phases is by using the studies of association in solution. There are multiple studies supporting that, in part of the cases, molecule association in solution and the formed prenucleation aggregates determine or directly influence the crystal structure obtained from these solutions [68].

**Figure 8:**
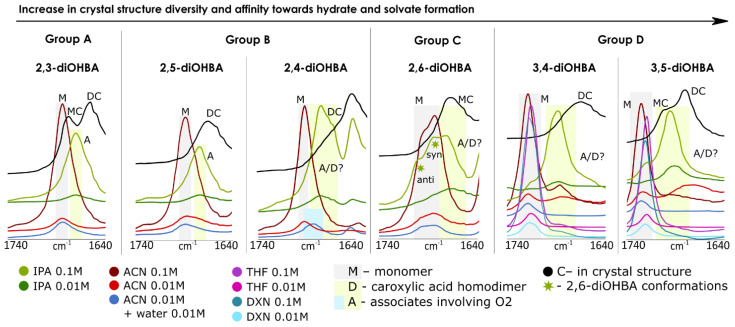
The recorded FTIR spectra of 0.1 M and 0.01 M solutions (in 2-propanol, acetonitrile, tetrahydrofuran, 1,4-dioxane) and solids of the most stable polymorph of all six diOHBA arranged based on the overall structure diversity of the compounds and the number of hydrates and solvates formed. Absorption bands are assigned to the most likely species present in the solution. Absorption bands corresponding to the 2,6-diOHBA conformers are labelled with asterisks.

**Figure 9:**
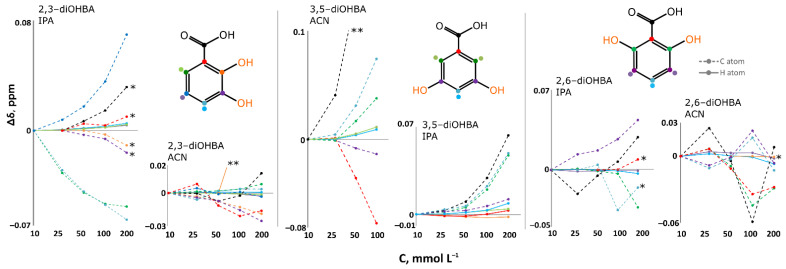
The ^1^H and ^13^C chemical shift dependence on concentration in the recorded NMR spectra of all 2,4-, 2,6-, and 3,5-diOHBA solutions in acetonitrile and 2-propanol (using a logarithmic scale for the concentration axis). In all graphs an equal scale is used. Points corresponding to the ^13^C chemical shift changes are joined by dotted lines, and those corresponding to ^1^H are joined by solid lines, with both lines being a guide for the eye. Color coding is used to assign each signal to a particular atom. One asterisk (*) indicates the signal is not detected for the lowest concentration solutions; two asterisks (**) indicate values continue to increase and goes outside of the showed chemical shift change range.

Measurements of FTIR spectra were carried out in acetonitrile (3,4- and 3,5-diOHBAs form acetonitrile solvate, while other compounds crystallize in the most stable neat form), in acetonitrile in the presence of water (to draw conclusions on the effect of the presence of water) and 2-propanol (none of the compounds form solvate, while evaporation in ambient conditions results in the formation of 3,4- and 3,5-diOHBA hydrates). Additional experiments with 3,4- and 3,5-diOHBAs were carried out in 1-4-dioxane (both compounds form solvate) and THF (3,5-diOHBA forms two different solvates). Among all the six compounds, 2,3- 2,6-, and 3,5-diOHBAs were chosen for further investigation using NMR spectroscopy, by studying acetonitrile-*d3* and 2-propanol-*d8* solutions. Both 2,3- and 3,5-diOHBAs were chosen as the least and the most prolific solid form formers (representing Group A and Group D, respectively), and 2,6-diOHBA (Group C) was chosen because this compound can exist in solution as a *syn* and/or *anti* conformer. Unfortunately, the O–H stretching region (3500–3000 cm^−1^), which could provide insight into interactions between hydroxyl groups and solvent molecules, could not be used since background noise strongly interfered and prevented detection of the respective absorption peaks. Thus, in the IR spectra, we focused on the region of the C=O antisymmetric stretch to determine the presence of carboxylic acid homodimers and other interactions altering the frequency of C=O stretching band in the solution. Figure 8 shows the C=O stretching region in all the recorded FTIR spectra; Figure 9 shows the chemical shift concentration dependence in NMR spectra.

For identification of the characteristic band positions, the FTIR spectra of the most stable polymorphs of diOHBAs (polymorph I of 2,3-, 3,4-, 3,5-diOHBAs and polymorph II of 2,4-, 2,5-, 2,6-diOHBAs) were recorded. In the crystal structures of all the *ortho*-substituted diOHBA there are intramolecular bonds S(6) (O3–H2^…^O2), and in most structures there are carboxylic acid homodimers R^2^_2_(8), with the only exception being 2,6-diOHBA, for which polymorph II contains an *anti*-conformer that does not form homodimers.Apart from the main stretch band exhibited by homodimers, a second stretch band is present for 2,3-diOHBA (Z′ = 2), corresponding to the only R_3_^3^(12) motif (O2^…^H2–O3) forming molecules, and for 3,5-diOHBA (Z′ = 2) corresponding to the molecules involved in chains C(7) (O2^…^H5–O4). Thus, based on the position of the observed bands, the C=O antisymmetric stretch region for these molecules was determined as 1740–1640 cm^−1^.

It should be noted that in Figure 8, the shift of the absorption bands in solution compared to that in the solid is caused by the differences in steric and electronic effects in both of these media; therefore, in solution, pure monomer peaks are expected at a higher wavelength than in the respective solid structures, where C=O is involved in stronger and less dynamic hydrogen bonding.

In FTIR spectra of pure acetonitrile solutions of 2,3-, 2,4- and 2,5-diOHBAs (compounds of Group A and B), only the absorption band of the monomers was observed, and thus no formation of carboxyl acid homodimers in the solution was detected. The addition of water has no observable effect on 2,3- and 2,5-diOHBAs in acetonitrile, whereas the addition of water slightly shifts the C=O stretching band of 2,4-diOHBA, indicating some involvement in association with water (note that from these three compounds only 2,4-diOHBA forms hydrate). NMR spectra of 2,3-diOHBA solutions in acetonitrile-*d3* support the conclusions from the FTIR spectra mentioned above; no notable systematic chemical shift changes in neither the ^13^C nor ^1^H spectra (except for the peaks of phenolic hydroxyl groups; see Figure S7 in the Supplementary Materials) in the considered concertation range indicates that no significant self-association of the compound molecules is occurring. In contrast, the peak positions in the FTIR spectra of the 2,3-, 2,4- and 2,5-diOHBA 2-propanol solutions indicate the formation of self-associates involving O2. Furthermore, for the solution of 2,4-diOHBA, there are multiple overlapping absorption bands, indicating the formation of multiple associates and/or even the existence of carboxylic acid homodimers in the solution. In the NMR spectra of the 2,3-diOHBA 2-propanol solution, an increase of the concentration introduces a downfield shift for C5, C7, and all the detectable H peaks, further confirming the formation of self-associates.

The interpretation of 2,6-diOHBA FTIR spectra in both acetonitrile and 2-propanol is complicated by the presence of both conformers in the solution, resulting in multiple overlapping peaks. In acetonitrile solution, only absorption bands that can be assigned to both conformers are detected, as the difference of ≈15 cm^−1^ between the experimental peaks well corresponds to the difference of frequencies for both monomers (14 cm^−1^) calculated in Gaussian 09 (Gaussian, Inc., Wallingford, CT, USA) [46]. The addition of water to the acetonitrile solution had no effect on the association. In contrast, in 2-propanol an additional peak can be clearly detected. As the position of this peak is at a lower wavelength, it is expected to belong to potential self-associates (most likely an associate linked by the interaction between carboxylic acid and the phenolic hydroxyl group or carboxyl acid homodimer). However, no clearly detectable chemical shift concentration dependence for 2,6-diOHBA peaks was observed in none of the recorded solution NMR spectra.

The FTIR spectra for both Group D compounds in the acetonitrile, tetrahydrofuran, and 1,4-dioxane solutions are very similar: along the absorption band corresponding to the monomers, a weak additional band that could not be clearly assigned to any particular species was detected. However, the relative intensity of this band with respect to the monomer band in 3,4-diOHBA acetonitrile solutions seems to increase with the dilution, and thus it could correspond to an associate linked by an interaction between carboxylic acid and a phenolic hydroxyl group or carboxyl acid homodimer. This assumption was additionally supported by the absence of this band in solution with added water, as such hydrogen bonded associates are less likely found in polar solvents since the highly polar water already hydrogen bonds to the solute hydroxyl groups [69]. In contrast, in the solutions of 2-propanol, the band corresponding to the monomers has very low relative intensity while a pronounced band in almost the same position as the previously described band in the acetonitrile solution is present. However, the relative intensity of this band with respect to the monomer band seems to be concentration independent and therefore likely corresponds to hydrogen-bonded associates with 2-propanol. In the NMR spectra of the 3,5-diOHBA acetonitrile and 2-propanol solution, there is a considerable downfield shift of all the detectable 3,5-diOHBA proton peaks as well as the C7, C2, C6, and C4 peaks, meaning that self-association is most likely occurring, and the number of associates increases by increasing the concentration of 3,5-diOHBA.

Overall, the results from both the FTIR and NMR spectra indicate that only Group D compounds form a carboxyl group involving associates in acetonitrile solution while in 2-propanol all the compounds experience self-association (formation of carboxylic acid–phenolic hydroxyl group associates or carboxylic acid homodimers) and/or forms associates in which the carboxylic group is bonded with 2-propanol.

### 3.4. Studies of Association of diOHBAs in Solutions Using Molecular Dynamics (MD) Simulations

In addition to the spectroscopic studies, we also investigated the behaviour of diOHBA molecules in solution using MD simulations using a simulation box representing solutions of 2,3-diOHBA (Group A), 2,4-diOHBA (Group B), 3,4-, and 3,5-diOHBAs (Group D) in acetonitrile, tetrahydrofuran, and 2-propanol as well as 2,6-diOHBA (Group C) in 2-propanol with a concentration of ≈0.15 M.

**Figure 10:**
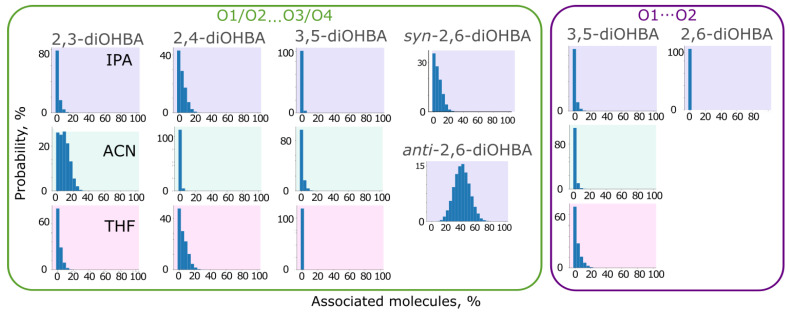
Selected graphs showing the probability of diOHBAs being involved in the formation of hydrogen bonded associates linked by carboxyl group–phenolic hydroxyl group interactions (O1/O2^…^O3/O4) and interactions between two carboxylic groups (O1^…^O2). All the graphs are given in Figures S8 and S9 in the Supplementary Materials.

Firstly, the obtained MD trajectories were analyzed by identifying the extent of self-association of diOHBA in solution by using the VMD HBond plugin (see Figures S8 and S9 in the Supplementary Materials and Figure 10 for selected graphs). In none of the simulations is the self-association extensive, except for the 2,6-diOHBA *anti*-conformer that exhibited a pronounced formation of carboxyl group–phenolic hydroxyl group (O1/O2^…^O3/O4) associates. This observation, however, disagrees with the observations from FTIR (in 2-propanol solutions, the absorption bands corresponding to species in which O2 is involved in associates were more pronounced than the band corresponding to the monomers) and NMR spectra (downfield shift associated with self-association in 2,3- and 3,5-diOHBA solutions). For all *ortho*-substituted diOHBAs, the most abundant associate in the simulations is the carboxyl group–phenolic hydroxyl group (O1/O2^…^O3/O4) associate, and the formation of associates involving hydrogen bond O1–H^…^O2 is insignificant. However, opposite abundances were observed for the non-*ortho*-substituted diOHBAs, for which the formation of O1/O2^…^O3/O4 associates is negligible.

Based on these results, we conclude that the solvent plays only a minor effect on the ability of diOHBAs to form self-associates. Although the carboxylic acid homodimer is the hydrogen bond motif found in most of the crystal structures of diOHBAs, the absence of notable formation carboxylic acid homodimers in all simulations indicates that this is the most efficient building block in the solid state, but its formation is not caused by its presence in the solution.

Further, visual inspection of every 1 ns of the obtained MD trajectories (in total 100 frames for each simulation) in 2-propanol solution confirmed that the majority of the diOHBA molecules exist as monomers. The most typical associates, as expected from the previously described graphs, are the carboxyl group–phenolic hydroxyl group (O1/O2^…^O3/O4) associates, particularly self-associates with hydrogen bond O2^…^H–O3/O4, also simultaneously forming interaction O1–H^…^O2, followed by associates linked by phenolic hydroxyl groups (O3/O4^…^O3/O4). Non-*ortho*-substituted diOHBAs (Group D) mostly formed associates linking carboxyl groups (O1^…^O2), often having only one O2^…^H–O1 bond or a deformed carboxylic acid homodimer, as well as in a smaller extent associates linked by carboxyl group–phenolic hydroxyl group interactions (O2^…^H–O4).

In all simulations, π^…^π stacking of diOHBA molecules to a various degree could also be observed, particularly for 2,3- and *anti*-2,6-diOHBA (Group A and C) while it was almost absent for 2,4-diOHBA (Group B). However, classically π^…^π stacked molecules are notably less common than molecules exhibiting π^…^π interactions resembling those found in the crystal structures and discussed in Section 3.1.

In the simulations, a majority of the molecules did not experience *syn*–*anti* conformation change. However, 2,6-diOHBA (Group C), 3,4-, and 3,5-diOHBAs (Group D) are characterized by the formation of different conformations associated with differences in the relative arrangement of carboxyl group (as discussed above). Furthermore, in simulations of both *syn* and *anti* 2,6-diOHBA and 3,5-diOHBA, the carboxyl group rotates rather freely without *syn*–*anti* conformation changes. This could additionally explain how the previously discussed carboxyl group relative arrangement in 3,4-diOHBA crystalline phases promotes the formation of hydrates rather than the nonsolvated phase.

From the trajectories of MD simulations, we can also see that all the considered solvents form hydrogen bonds with diOHBAs, with acetonitrile and tetrahydrofuran both acting only as hydrogen bond acceptors, and also 2-propanol mostly being involved as a hydrogen bond acceptor (O_IPA_^…^H–O1/O3/O4) and to a smaller extent also as a donor (O_IPA_–H^…^O2) (see Figures S8 and S9 in the Supplementary Materials).

**Figure 11:**
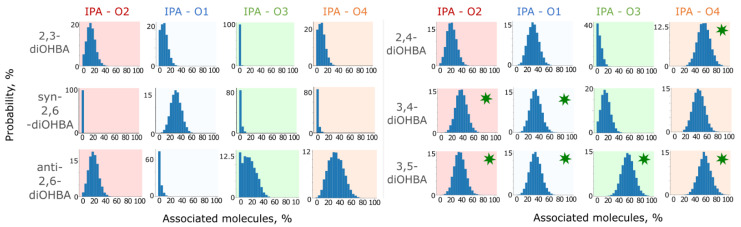
Probability that the given relative number of diOHBAs will be involved in a hydrogen bond between the specified O atoms of diOHBA and the hydroxyl group of 2-propanol (IPA). Interactions with diOHBA O atoms that are not involved in intramolecular hydrogen bonds are highlighted by green stars.

Results from simulations in 2-propanol were examined in more details (see Figure 11). A comparison of the association probability in 2-propanol of both *ortho*-substituted and the non-*ortho*-substituted diOHBAs indicate that the intramolecular bond O3–H^…^O2 slightly reduces the number of molecules forming hydrogen bond O2^…^H_IPA_ while, as expected, heavily affecting the ability of O3 to form a hydrogen bond with 2-propanol molecules. The exception is the hydroxyl groups of *anti*-2,6-diOHBA, which despite the intramolecular bonds O3–H3^…^O2 and O1–H1^…^O4, can form hydrogen bonds with 2-propanol as well as 3,4-diOHBA can. The amount of interaction with O1–H^…^O_IPA_ is not heavily affected either by the formation of a single intramolecular bond (in 2,3-diOHBA and 2,4-diOHBA) or by the identity of the solvent, although the number of diOHBA molecules involved in the O1–H^…^N_ACN_ bond is lower than those involved in the O1–H^…^O_solvent_.

In the case of the diOHBAs of Group D having two hydroxyl groups that are extremely good hydrogen bond donors and good acceptors, the formation of associates with 2-propanol is inevitable: the probability of formation of interaction O3/O4^…^O2_solvent_ for both compounds is considerably higher than that for *ortho*-substituted diOHBAs. Furthermore, this observation agrees with the conclusions from the analysis of ESP surfaces: both free 3,5-diOHBA hydroxyl groups have equal ESP extrema values, thus resulting in equal association probability. However, the intramolecular bond between the phenolic hydroxyl groups slightly reduces the probability of association with solvent for 3,4-diOHBA; the intramolecular bond O3–H^…^O4 affects the ability of O3 to interact and extensively bond with the 2-propanol molecules, while O4 can bond as easily as the free hydroxyl groups of 2,4- and 3,5-diOHBAs.

**Figure 12:**
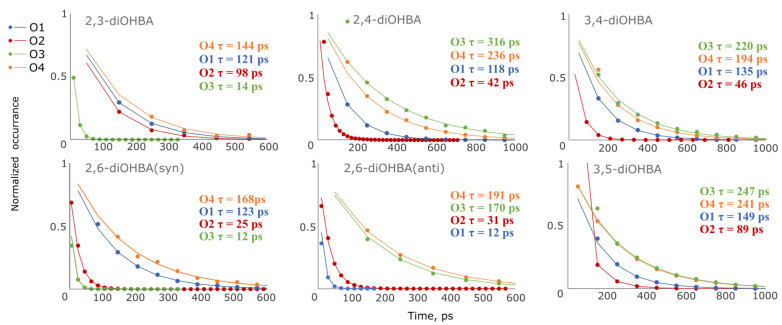
Distribution of lifetime of hydrogen-bonded diOHBA-2-propanol pairs formed by different diOHBA atoms obtained from trajectories of MD simulations. The mean lifetimes are calculated by an exponentially decaying function and in each case are given on the right. The occurrence was normalized by the height constant K of the theoretical equation.

Besides the characterization of the relative number of molecules involved in the hydrogen bonding with the solvent, we additionally investigated the distribution of the lifetime of hydrogen-bonded solute–solvent molecule pairs in 2-propanol using the obtained MD trajectories. The mean lifetime of the solute–solvent associates was obtained by fitting the solvent molecule escape time distribution to an exponentially decaying function, see Figure 12. It should be noted that the previously described conformation changes affect the mean lifetime for both phenolic hydroxyl groups of 2,6-, 3,4-, and 3,5-diOHBA, therefore averaging out the difference between both these groups.

For 2,4-, 3,4-, 3,5-, and *anti*-2,6-diOHBAs, the mean lifetime of solute–solvent associates formed by the phenolic hydroxyl group are mutually comparable (170–316 ps, averaging at 227 ps), and neither the intramolecular bond O3–H^…^O4 in 3,4-diOHBA nor the O2^…^H–O3 in 2,4-diOHBA has a notable effect on the mean lifetime of the given bonds. In contrast, for 2,3- and *syn*-2,6-diOHBAs, the mean lifetime of solute–solvent associates formed by O4 is notably lower (144 and 168 ps) and negligible (12–14 ps) when formed by O3. Furthermore, for compounds that have known hydrated phases (2,4-, 3,4-,3,5- and *anti*-2,6-diOHBAs), the mean lifetime of solute–solvent associates formed by the phenolic hydroxyl group is always higher than that of the solute–solvent associates formed by the carboxyl group.

The mean lifetime of the solute–solvent associates formed by the carboxylic group in all *syn* conformation diOHBAs are close in value (range of 118–149 ps for O1 and 25–98 ps for O2) and are only slightly reduced by the formation of the intermolecular bond O2^…^H–O3. Additionally, the ESP surface extrema values on O1 and O2 discussed earlier in this paper also do not differ significantly among these compounds. However, we can also see that the mean lifetime of the solute–solvent associate formed by phenolic hydroxyl groups does not strongly correlate with the probability of the formation of such associates (Figure 11), nor does it have a clear connection to the ESP surfaces (Figure 7).

### 3.5. Summary of the Link Between Solvate Formation, Crystal Structures, and Association in Solution

Summarizing the results from all the employed methods, the intramolecular hydrogen bond O2^…^H–O3 can be identified as the main factor that determines the diversity of solid phases formed by the diOHBAs. In Table 2, we distinguished the diOHBAs based on the presence of an O2^…^H–O3 hydrogen bond formed by *ortho*-substituted isomers, and we summarized the observed unique characteristics and common features (according to each employed method).

## 4. Conclusions

In summary, the propensity of diOHBAs to form different solid forms was determined by hydrogen bonds formed by phenolic hydroxyl groups, while carboxyl groups in most of the solvated and nonsolvated diOHBA crystal structures formed classical carboxyl acid homodimers R^2^_2_(8). The propensity to form different solid forms is notably different, as the 2,3-diOHBA tends to form only the most stable polymorph, whereas 3,4-diOHBA and 3,5-diOHBA form numerous solvated forms; based on this feature we classified diOHBAs in 4 groups. Despite the large number of solvates formed by 3,5-diOHBA with different solvents, the structural diversity in the solvates was limited, given that in all the solvates, solvent molecules were situated in channels enclosed by ring-like hydrogen bond motifs formed by two R^2^_2_(8) dimers and two additional linker diOHBA molecules.

Studies of association in solution demonstrated that the observed higher abundance of phenolic hydroxyl group associates can be linked to a higher solvate formation propensity, as these features are exhibited by diOHBAs having no hydroxyl group in *ortho* position. This is because these diOHBAs cannot form the intramolecular bond O2^…^H–O3, which would interfere with the formation of the phenolic hydroxyl group involving self-associates in solution as observed for the *ortho*-substituted diOHBAs. Additionally, the non-*ortho*-substituted diOHBAs have an even distribution of ESP extrema, resulting in the formation of intermolecular interactions, allowing for the incorporation of guest molecules.

## Figures and Tables

**Table 1:** Summary of the results of the performed solid form screening of diOHBAs from selected solvents and classification into groups according to exhibited propensity to form solid phases. Used abbreviations: tetrahydrofuran (THF), 1,4-dioxane (DXN), acetonitrile (ACN), 2-propanol (IPA), hemihydrate (HH), monohydrate (MH), solvate (S).

Solvent	THF	DXN	ACN	IPA	Water	Group
Compound						
2,3-diOHBA	I	I	I	I	I	Group A
2,5-diOHBA	II	II	II	I + II	II	Group B
2,4-diOHBA	I/II	II + I	II	II	HH	
2,6-diOHBA	MH/ II + MH	MH/ II + MH	MH+II	II	MH	Group C
3,4-diOHBA	I + MH I + MH II	S_0.5DXN_	S_ACN_ + MH II /S_ACN_	MH I	MH I	Group D
3,5-diOHBA	S_0.25THF_MH /S_0.5THF_	S_DXN_	S_ACN_	I + HH	MH I	

**Table 2:** Summary of the identified unique characteristics and common features of diOHBAs.

*Ortho*-Substituted diOHBA	Non*-Ortho*-Substituted diOHBA
**Crystal form landscape**	
Low propensity to form solvates; can be divided into Group A, Group B (for both groups mostly the most stable polymorph was obtained) and Group C (prone to form hydrate).	Readily forms hydrates and solvates; nonsolvated phases are complicated to obtain in crystallization (Group D compounds).
**Crystal structure evaluation**	
The most efficient building blocks are classical carboxyl acid homodimer R^2^_2_(8) and ring-like hydrogen bond (RHB) motifs involving six molecules (i.e., two R^2^_2_(8) dimers and two additional linker molecules). Molecules in general pack efficiently; packing index differences are low and does not affect the solvate formation.	
If able, phenolic hydroxyl groups form infinite hydrogen-bonded chains, which stabilize the structures and the incorporation of guest molecules in the structure is hindered.	Ring-like hydrogen bond motifs are essential for solvate formation. In case of 3,5-diOHBA, guest molecules stabilize the structures.
**Electrostatic potential surfaces of diOHBA**	
Intramolecular bond O2^…^H–O3 causes an uneven distribution of ESP extrema in the molecule.	ESP extrema values on phenolic hydroxy and carboxyl groups does not significantly differ, ESP extrema are evenly distributed across the molecule.
**Spectroscopy studies of association**	
Most probable associates are carboxylic acid–phenolic hydroxyl group self-associates, carboxylic acid homodimers, and/or associates in which carboxylic group is bonded with solvent molecule.	
Carboxyl group involving associates are present only in 2-propanol solutions.	Carboxyl group involving associates are present in acetonitrile and 2-propanol solutions.
**Molecular dynamics simulations**	
Absence of notable amount of carboxylic acid homodimers in any conditions. Intramolecular hydrogen bond has no notable effect on the mean lifetime of solute–solvent associates formed by the phenolic hydroxyl group. For hydrate-forming compounds, the mean lifetime of solute–solvent associates formed by phenolic hydroxyl group is always higher than that of the solute–solvent associates formed by carboxyl group.	
In simulations, the most abundant are the carboxyl group–phenolic hydroxyl group associates, followed by the phenolic hydroxyl group associates. The intramolecular bond O3–H^…^O2 heavily affects the abundance of associates formed by the hydrogen bond between the phenolic hydroxyl group (O3) and solvent molecules.	In simulations, the most abundant are carboxyl group self-associates. The probability of the formation of a phenolic hydroxyl group–solvent interaction is considerably higher than that exhibited by *ortho*-substituted diOHBAs. The intramolecular bond between the phenolic hydroxyl groups has almost no effect on the probability of association with the solvent.

## Data Availability

CCDC 2077764-2077766 contain the supplementary crystallographic data for this paper. These data can be obtained free of charge via www.ccdc.cam.ac.uk/data_request/cif, or by emailing data_request@ccdc.cam.ac.uk, or by contacting The Cambridge Crystallographic Data Centre, 12 Union Road, Cambridge CB2 1EZ, UK.

## Supplementary Materials

The following are available online at https://www.mdpi.com/article/10.3390/pharmaceutics13050734/s1. Figure S1: DSC-TG analysis of nonsolvated and undescribed solvated phases of 2,3-diOHBA, Figure S2: DSC-TG analysis of nonsolvated and undescribed solvated phases of 2,5-diOHBA, Figure S3: DSC-TG analysis of nonsolvated and undescribed solvated phases of 2,4-diOHBA, Figure S4: DSC-TG analysis of nonsolvated and undescribed solvated phases of 2,6-diOHBA, Figure S5: DSC-TG analysis of nonsolvated and undescribed solvated phases of 3,4-diOHBA, Figure S6: DSC-TG analysis of nonsolvated and undescribed solvated phases of 3,5-diOHBA, Figure S7: The ^1^H and ^13^C chemical shift dependence on concentration in the recorded NMR spectra of all 2,4-, 2,6- and 3,5-diOHBA solutions in acetonitrile and 2-propanol, Figure S8: Probability for the selected Group A (2,3-diOHBA), Group B (2,4-diOHBA) and Group C (2,6-diOHBA, two conformations) compounds that the given relative amount of dOHBAs will be involved in a hydrogen bond between the specified O atoms of diOHBA and specified O atoms of diOHBA and hydroxyl group of 2-propanol (IPA), nitrile group of acetonitrile (ACN) and O atom of tetrahydrofuran (THF), Figure S9: Probability for Group D (3,4-, 3,5-diOHBA) compounds that the given relative amount of dOHBAs will be involved in a hydrogen bond between the specified O atoms of diOHBA and specified O atoms of diOHBA and hydroxyl group of 2-propanol (IPA), nitrile group of acetonitrile (ACN) and O atom of tetrahydrofuran (THF), Figure S10: Hydrogen bond motifs in crystal structures of all known nonsolvated phases of 2,3-diOHBA, Figure S11: Hydrogen bond motifs in crystal structures of all known nonsolvated phases of 2,5-diOHBA, Figure S12: Hydrogen bond motifs in crystal structures of all known nonsolvated and hydrated phases of 2,5-diOHBA, Figure S13: Hydrogen bond motifs in crystal structures of all known nonsolvated and hydrated phases of 2,6-diOHBA, Figure S14: Hydrogen bond motifs in crystal structures of all known nonsolvated and hydrated phases of 3,4-diOHBA, Figure S15: Hydrogen bond motifs in crystal structures of all known nonsolvated and hydrated phases of 3,5-diOHBA, Figure S16: The identified types of 3,5-diOHBA solvated forms based on the RHB motif in their structure and hydrogen bond motifs in crystal structure of 3,5-diOHBA 1,4-dioxane solvate, Figure S17: Phase identification of selected obtained products using PXRD patterns simulated from the crystal structures deposited in the CSD, Table S1: Summary of the results obtained in solid form screening from common solvents by using cooling crystallization and crystallization by slow evaporation in ambient conditions, Table S2: Crystal data and structure refinement details for newly characterized solvates, Table S3: Summary of structural information on nonsolvated and selected solvated crystalline phases–crystallographic information, calculated packing index and lattice energy, obtained experimental melting enthalpies (nonsolvated phases) and the main hydrogen bond motifs, Table S4: Hydrogen bond geometric parameters and motifs in crystal structures of all known nonsolvated phases of 2,3-diOHBA and corresponding hydrogen bond parameters. Table S5: Hydrogen bond geometric parameters and motifs in crystal structures of all known nonsolvated phases of 2,5-diOHBA and corresponding hydrogen bond parameters, Table S6: Hydrogen bond geometric parameters and motifs in crystal structures of all known nonsolvated phases of 2,4-diOHBA and corresponding hydrogen bond parameters, Table S7: Hydrogen bond geometric parameters and motifs in crystal structures of all known hydrated phases of 2,4-diOHBA and corresponding hydrogen bond parameters, Table S8: Hydrogen bond geometric parameters and motifs in crystal structures of all known nonsolvated and hydrated phases of 2,6-diOHBA and corresponding hydrogen bond parameters, Table S9: Hydrogen bond geometric parameters and motifs in crystal structures of all known nonsolvated and hydrated phases of 3,4-diOHBA and corresponding hydrogen bond parameters, Table S10: Hydrogen bond geometric parameters and motifs in crystal structures of all known nonsolvated and hydrated phases of 3,5-diOHBA and corresponding hydrogen bond parameters.

## References

[1] Cruz-Cabeza A.J., Reutzel-Edens S.M., Bernstein J. (2015). Facts and fictions about polymorphism. Chem. Soc. Rev..

[2] Healy A.M., Worku Z.A., Kumar D., Madi A.M. (2017). Pharmaceutical solvates, hydrates and amorphous forms: A special emphasis on cocrystals. Adv. Drug Deliv. Rev..

[3] Corpinot M.K., Bučar D.K. (2019). A Practical Guide to the Design of Molecular Crystals. Cryst. Growth Des..

[4] Bhardwaj R.M., Price L.S., Price S.L., Reutzel-Edens S.M., Miller G.J., Oswald I.D.H., Johnston B.F., Florence A.J. (2013). Exploring the experimental and computed crystal energy landscape of olanzapine. Cryst. Growth Des..

[5] Braun D.E., Ardid-Candel M., D’Oria E., Karamertzanis P.G., Arlin J.B., Florence A.J., Jones A.G., Price S.L. (2011). Racemic naproxen: A multidisciplinary structural and thermodynamic comparison with the enantiopure form. Cryst. Growth Des..

[6] Hulme A.T., Price S.L., Tocher D.A. (2005). A new polymorph of 5-fluorouracil found following computational crystal structure predictions. J. Am. Chem. Soc..

[7] Price S.L., Braun D.E., Reutzel-Edens S.M. (2016). Can computed crystal energy landscapes help understand pharmaceutical solids?. Chem. Commun..

[8] Braun D.E., Karamertzanis P.G., Price S.L. (2011). Which, if any, hydrates will crystallise? Predicting hydrate formation of two dihydroxybenzoic acids. Chem. Commun..

[9] Braun D.E., Griesser U.J. (2016). Why Do Hydrates (Solvates) Form in Small Neutral Organic Molecules? Exploring the Crystal Form Landscapes of the Alkaloids Brucine and Strychnine. Cryst. Growth Des..

[10] Xin D., Gonnella N.C., He X., Horspool K. (2019). Solvate Prediction for Pharmaceutical Organic Molecules with Machine Learning. Cryst. Growth Des..

[11] Musil F., De S., Yang J., Campbell J.E., Day G.M., Ceriotti M. (2018). Machine learning for the structure-energy-property landscapes of molecular crystals. Chem. Sci..

[12] Takieddin K., Khimyak Y.Z., Fábián L. (2016). Prediction of Hydrate and Solvate Formation Using Statistical Models. Cryst. Growth Des..

[13] Cole J.C., Raithby P.R., Taylor R. (2021). Prior Likelihoods and Space-Group Preferences of Solvates. Cryst. Growth Des..

[14] Werner J.E., Swift J.A. (2021). Organic solvates in the Cambridge Structural Database. CrystEngComm.

[15] Cruz-Cabeza A.J., Feeder N., Davey R.J. (2020). Open questions in organic crystal polymorphism. Commun Chem.

[16] Aminpour M., Montemagno C., Tuszynski J.A. (2019). An overview of molecular modeling for drug discovery with specific illustrative examples of applications. Molecules.

[17] Scheraga H.A., Khalili M., Liwo A. (2007). Protein-folding dynamics: Overview of molecular simulation techniques. Annu. Rev. Phys. Chem..

[18] Jalili S., Amani P. (2014). Molecular dynamics simulation study of solvation effects of water and trifluoroethanol on gamma-aminobutyric acid (GABA). J. Mol. Liq..

[19] Salvalaglio M., Perego C., Giberti F., Mazzotti M., Parrinello M. (2015). Molecular-dynamics simulations of urea nucleation from aqueous solution. Proc. Natl. Acad. Sci. USA.

[20] Polêto M.D., Grisci B.I., Dorn M., Verli H. (2020). ConfID: An analytical method for conformational characterization of small molecules using molecular dynamics trajectories. Bioinformatics.

[21] Boothroyd S., Kerridge A., Broo A., Buttar D., Anwar J. (2018). Why Do Some Molecules Form Hydrates or Solvates?. Cryst. Growth Des..

[22] Bērziņš A., Kons A., Saršūns K., Belyakov S., Actiņš A. (2020). On the rationalization of formation of solvates: Experimental and computational study of solid forms of several nitrobenzoic acid derivatives. Cryst. Growth Des..

[23] Bērziņš A., Zvaniņa D., Trimdale A., Bērziņš A., Zvaniņa D., Trimdale A. (2018). Detailed Analysis of Packing Efficiency Allows Rationalization of Solvate Formation Propensity for Selected Structurally Similar Organic Molecules. Cryst. Growth Des..

[24] Braun D.E., McMahon J.A., Koztecki L.H., Price S.L., Reutzel-Edens S.M. (2014). Contrasting polymorphism of related small molecule drugs correlated and guided by the computed crystal energy landscape. Cryst. Growth Des..

[25] Case D.H., Srirambhatla V.K., Guo R., Watson R.E., Price L.S., Polyzois H., Cockcroft J.K., Florence A.J., Tocher D.A., Price S.L. (2018). Successful Computationally Directed Templating of Metastable Pharmaceutical Polymorphs. Cryst. Growth Des..

[26] Horneffer V., Dreisewerd K., Lüdemann H.C., Hillenkamp F., Läge M., Strupat K. (1999). Is the incorporation of analytes into matrix crystals a prerequisite for matrix-assisted laser desorption/ionization mass spectrometry? A study of five positional isomers of dihydroxybenzoic acid. Int. J. Mass Spectrom..

[27] Bērziņš A., Actiņš A. (2016). Why Do Chemically Similar Pharmaceutical Molecules Crystallize in Different Structures: A Case of Droperidol and Benperidol. Cryst. Growth Des..

[28] McGregor L., Rychkov D.A., Coster P.L., Day S., Drebushchak V.A., Achkasov A.F., Nichol G.S., Pulham C.R., Boldyreva E.V. (2015). A new polymorph of metacetamol. CrystEngComm.

[29] Trotta J.T., Zeidan T.A., Tilak P.A., Foxman B.M., Almarsson Ö., Oliveira M.A., Chiarella R.A., Hickey M.B., Remenar J.F. (2020). Aripiprazole and Dehydro-Aripiprazole Solid Solutions: Crystalline Combinations of Drug and Active Metabolite in Tailored Compositions. Cryst. Growth Des..

[30] Srirambhatla V.K., Guo R., Price S.L., Florence A.J. (2016). Isomorphous template induced crystallisation: A robust method for the targeted crystallisation of computationally predicted metastable polymorphs. Chem. Commun..

[31] Zeidan T.A., Trotta J.T., Tilak P.A., Oliveira M.A., Chiarella R.A., Foxman B.M., Almarsson Ö., Hickey M.B. (2016). An unprecedented case of dodecamorphism: The twelfth polymorph of aripiprazole formed by seeding with its active metabolite. CrystEngComm.

[32] Okabe N., Kyoyama H. (2001). 2,3-Dihydroxybenzoic acid. Acta Crystallogr. Sect. E Struct. Reports Online.

[33] Haisa M., Kashino S., Hanada S.-I., Tanaka K., Okazaki S., Shibagaki M. (1982). The structures of 2-hydroxy-5-methylbenzoic acid and dimorphs of 2,5-dihydroxybenzoic acid. Acta Crystallogr. Sect. B Struct. Crystallogr. Cryst. Chem..

[34] Braun D.E., Karamertzanis P.G., Arlin J.B., Florence A.J., Kahlenberg V., Tocher D.A., Griesser U.J., Price S.L. (2011). Solid-state forms of β-resorcylic acid: How exhaustive should a polymorph screen be?. Cryst. Growth Des..

[35] Gdaniec M., Gilski M., Denisov G.S. (1994). γ-Resorcylic acid, its monohydrate and its pyridinium complex. Acta Crystallogr. Sect. C Cryst. Struct. Commun..

[36] MacGillivray L.R., Zaworotko M.J. (1994). Crystal and molecular structure of 2,6-dihydroxybenzoic acid. J. Chem. Crystallogr..

[37] Mazurek J., Dova E., Helmond R. (2007). 3,4-Dihydroxybenzoic acid acetonitrile solvate at 120 K. Acta Crystallogr. Sect. E Struct. Reports Online.

[38] Sarma B., Sanphui P., Nangia A. (2010). Polymorphism in isomeric dihydroxybenzoic acids. Cryst. Growth Des..

[39] Varughese S., Desiraju G.R. (2010). Using water as a design element in crystal engineering. Host-guest compounds of hydrated 3,5-dihydroxybenzoic acid. Cryst. Growth Des..

[40] Bērziņš A., Trimdale A., Kons A., Zvaniņa D. (2017). On the formation and desolvation mechanism of organic molecule solvates: A structural study of methyl cholate solvates. Cryst. Growth Des..

[41] Galabov B., Bobadova-Parvanova P., Ilieva S., Dimitrova V. (2003). The electrostatic potential at atomic sites as a reactivity index in the hydrogen bond formation. Proc. J. Mol. Struct.: THEOCHEM.

[42] Bajpai A., Scott H.S., Pham T., Chen K.J., Space B., Lusi M., Perry M.L., Zaworotko M.J. (2016). Towards an understanding of the propensity for crystalline hydrate formation by molecular compounds. IUCrJ.

[43] Davey R.J., Dent G., Mughal R.K., Parveen S. (2006). Concerning the relationship between structural and growth synthons in crystal nucleation: Solution and crystal chemistry of carboxylic acids as revealed through IR spectroscopy. Cryst. Growth Des..

[44] Hansen P.E., Spanget-Larsen J. (2017). NMR and IR investigations of strong intramolecular hydrogen bonds. Molecules.

[45] Bobrovs R., Drunka L., Auzins A.A., Jaudzems K., Salvalaglio M. (2020). Polymorph-Selective Role of Hydrogen Bonding and π-π Stacking in p-Aminobenzoic Acid Solutions. Cryst. Growth Des..

[46] Frisch Æ., Plata R.E., Singleton D.A. (2009). Gaussian 09W Reference. J. Am. Chem. Soc..

[47] Di Tommaso D. (2013). The molecular self-association of carboxylic acids in solution: Testing the validity of the link hypothesis using a quantum mechanical continuum solvation approach. CrystEngComm.

[48] Lu T., Chen F. (2012). Multiwfn: A multifunctional wavefunction analyzer. J. Comput. Chem..

[49] Humphrey W., Dalke A., Schulten K. (1996). VMD: Visual molecular dynamics. J. Mol. Graph..

[50] Spek A.L. (2009). Structure validation in chemical crystallography. Acta Crystallogr. Sect. D Biol. Crystallogr..

[51] Mackenzie C.F., Spackman P.R., Jayatilaka D., Spackman M.A. (2017). CrystalExplorer model energies and energy frameworks: Extension to metal coordination compounds, organic salts, solvates and open-shell systems. IUCrJ.

[52] Kashinski D.O., Chase G.M., Nelson R.G., Di Nallo O.E., Scales A.N., Vanderley D.L., Byrd E.F.C. (2017). Harmonic Vibrational Frequencies: Approximate Global Scaling Factors for TPSS, M06, and M11 Functional Families Using Several Common Basis Sets. J. Phys. Chem. A.

[53] Wang J., Wolf R.M., Caldwell J.W., Kollman P.A., Case D.A. (2004). Development and testing of a general Amber force field. J. Comput. Chem..

[54] (2019). AMBER Amber 2019 Reference Manual. https://ambermd.org/doc12/Amber19.pdf.

[55] Caleman C., Van Maaren P.J., Hong M., Hub J.S., Costa L.T., Van Der Spoel D. (2012). Force field benchmark of organic liquids: Density, enthalpy of vaporization, heat capacities, surface tension, isothermal compressibility, volumetric expansion coefficient, and dielectric constant. J. Chem. Theory Comput..

[56] Van der Spoel D., van Maaren P.J., Caleman C. (2012). GROMACS molecule & liquid database. Bioinformatics.

[57] Van Der Spoel D., Lindahl E., Hess B., Groenhof G., Mark A.E., Berendsen H.J.C. (2005). GROMACS: Fast, flexible, and free. J. Comput. Chem..

[58] Parrinello M., Rahman A. (1981). Polymorphic transitions in single crystals: A new molecular dynamics method. J. Appl. Phys..

[59] Bussi G., Donadio D., Parrinello M. (2007). Canonical sampling through velocity rescaling. J. Chem. Phys..

[60] Tribello G.A., Bonomi M., Branduardi D., Camilloni C., Bussi G. (2014). PLUMED 2: New feathers for an old bird. Comput. Phys. Commun..

[61] Bonomi M., Bussi G., Camilloni C., Tribello G.A., Banáš P., Barducci A., Bernetti M., Bolhuis P.G., Bottaro S., Branduardi D. (2019). Promoting transparency and reproducibility in enhanced molecular simulations. Nat. Methods.

[62] Adam M.S., Gutmann M.J., Leech C.K., Middlemiss D.S., Parkin A., Thomas L.H., Wilson C.C. (2010). Stability and cooperativity of hydrogen bonds in dihydroxybenzoic acids. New J. Chem..

[63] Parkin A., Adam M., Cooper R.I., Middlemiss D.S., Wilson C.C. (2007). Structure and hydrogen bonding in 2,4-dihydroxybenzoic acid at 90, 100, 110 and 150 K; a theoretical and single-crystal X-ray diffraction study. Acta Crystallogr. Sect. B Struct. Sci..

[64] Sridhar B. (2015). Synthon preference in a hydrated β-resorcylic acid structure and its cocrystal with thymine. Acta Crystallogr. Sect. C Struct. Chem..

[65] Price C.P., Glick G.D., Matzger A.J. (2006). Dissecting the behavior of a promiscuous solvate former. Angew. Chemie—Int. Ed..

[66] Jensen T.T., Hall C.L., Potticary J., Andrusenko I., Gemmi M., Hall S.R. (2019). An experimental and computational study into the crystallisation propensity of 2nd generation sulflower. Chem. Commun..

[67] Rychkov D.A., Hunter S., Kovalskii V.Y., Lomzov A.A., Pulham C.R., Boldyreva E.V. (2016). Towards an understanding of crystallization from solution. DFT studies of multi-component serotonin crystals. Comput. Theor. Chem..

[68] Davey R.J., Schroeder S.L.M., Ter Horst J.H. (2013). Nucleation of organic crystals—A molecular perspective. Angew. Chemie—Int. Ed..

[69] Bux K., Moin S.T. (2020). Solvation of cholesterol in different solvents: A molecular dynamics simulation study. Phys. Chem. Chem. Phys..

