# Supplementary material for: Combined Use of Structure Analysis, Studies of Molecular Association in Solution, and Molecular Modelling to Understand the Different Propensities of Dihydroxybenzoic Acids to Form Solid Phases
Source: Pharmaceutics. 2021 May 16;13(5):734. doi: 10.3390/pharmaceutics13050734 (PMC8156891; doi:10.3390/pharmaceutics13050734)
Supplement: Supplementary file 1 [file pharmaceutics-13-00734-s001.zip › pharmaceutics-1204668-supplementary.pdf]

pharmaceutics

MDPI

Supplementary Materials: Combined Use of Structure Analysis, Studies of Molecular Association in Solution, and Molecular Modelling to Understand the Different Propensities of Dihydroxybenzoic Acids to Form Solid Phases

Aija Trimdale, Anatoly Mishnev and Agris Bērziņš

Table S1. Summary of the results obtained in solid form screening from common solvents by using cooling crystallization and crystallization by slow evaporation in ambient conditions.

Compound;<br>Solvent	2,3-diOHBA	2,5-diOHBA	2,4-diOHBA	2,6-diOHB<br>A	3,4-diOHBA	3,5-diOHBA
Group	Group A	Group B	Group B	Group C	Group D	Group D
Obtained known nonsolvated and hydrated phases and their respected refcodes	I (CACDAM) II (CACDAM01)	I (BESKAL01) II (BESKAL08)	I (ZZZEEU08) II (ZZZEEU04) HH (QIVTUK)	I (LEZJAB01) II (LEZJAB) MH (LEZJEF)	I (WUYNUA) MH I (BIJDON03) MH II (BIJDON04)	I (WUYPOW01)
Initial form used	I	II	II	II	I	I
Acetone	I	II + I	II	MH	MH I + MH II	$S_{0.2\ acetone}$ MH (OKEKEV)
Tetrahydrofuran THF	I	II	I / II	MH	I + MH I + MH II	$S_{0.25\ THF}$MH (WUYPIQ) / $S_{0.5\ THF}$
Acetonitrile ACN	I	II	II	MH + II	S_{ACN} (EDU-WUW)/S_{ACN} + MH II	S_{ACN}
1,4-dioxane DXN	I	II	II + I	MH	$S_{0.5\ DXN}$/$S_{0.5\ DXN}$+MH I	S_{DXN} (WUYPEM)
Nitromethane NM	I + II	II + I	I	I + II + MH	MH II	I
Methanol MeOH	I + II	II + I	II / HH	MH + II	MH I	? + I
Ethanol EtOH	I	II	II + HH	MH	MH I	? + I
2-propanol IPA	I	I + II	II + I	II	MH I	I + HH (OKEMAT)
Ethyl acetate EtOAc	II / I	II + I	II	MH + II	MH I	$S_{0.3\ EtOAC}$ MH I (OKEKAR)
Methyl ethyl ketone MEK	I	I / I + II	I + HH	II + MH	MH I /? + MH I	$S_{?\ MEK}$?H
Acetophenone PhAc	I	II	S_{PhAc}	$S_{0.5\ PhAc}$	$S_{0.2\ PhAc}$ +MH I/$S_{0.2\ PhAc}$	$S_{?\ PhAc}$?H
Toluene	II + I	–	–	I	–	I
Benzyl alcohol BnOH	I	II + I	$S_{0.2\ BnOH}$ or MH II	–	MH I	–
Acetic acid AcOH	I	S_{AcOH} (YOVVOW)	S_{AcOH}	II + MH	S_{AcOH}/I + S_{AcOH}	$S_{?\ AcOH}$?H
Formic acid	I	II	II + I	MH	MH I	I
Propionic acid	I	II	II + I	MH	MH I	I

Compound; Solvent	2,3-diOHBA	2,5-diOHBA	2,4-diOHBA	2,6-diOHBA	3,4-diOHBA	3,5-diOHBA
DMSO	$S_{?\ DMSO}$	S_{DMSO}	S_{DMSO} (UNAYOY)	–	S_{DMSO}	$S_{0.7\ DMSO}$ HH (OKEJOE)
DMA	$S_{1.5\ DMA}$	II	$S_{2\ DMA}$	S_{DMA}	$S_{0.7\ DMA}$	$S_{?\ DMA}$?H
DMF	I	S_{DMF}	II + I	–	$S_{?\ DMF}$ + MH I	–
Water	I	II	HH / HH + II	MH	MH I	MH (disordered)

Highlighted –previously undescribed solvated phases were obtained for which thermal analysis data were also obtained.

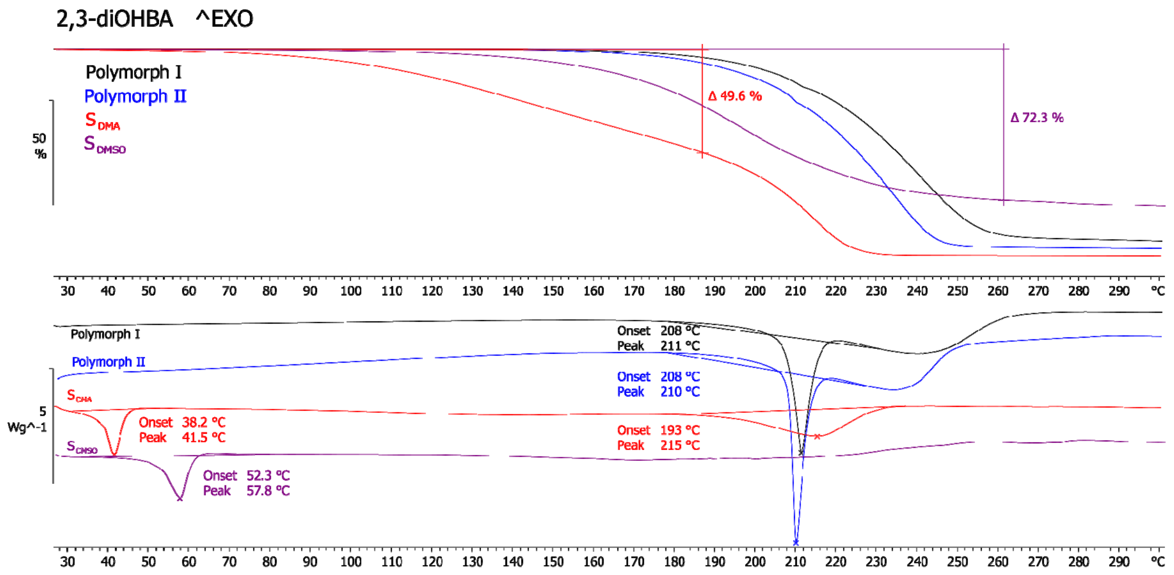

Figure S1. DSC-TG analysis of nonsolvated and undescribed solvated phases of 2,3-diOHBA.

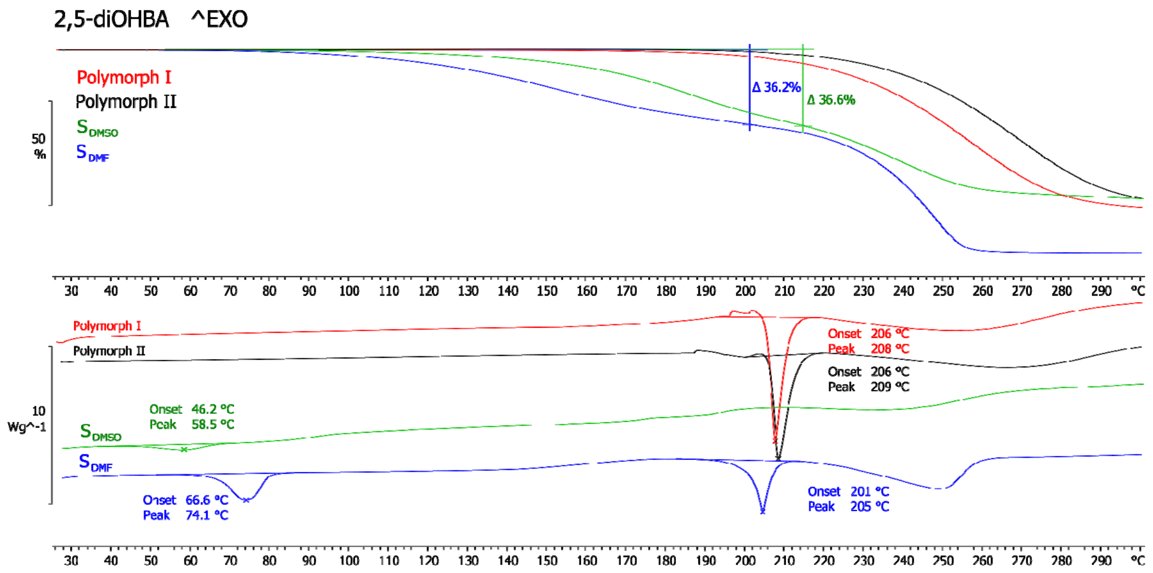

Figure S2. DSC-TG analysis of nonsolvated and undescribed solvated phases of 2,5-diOHBA.

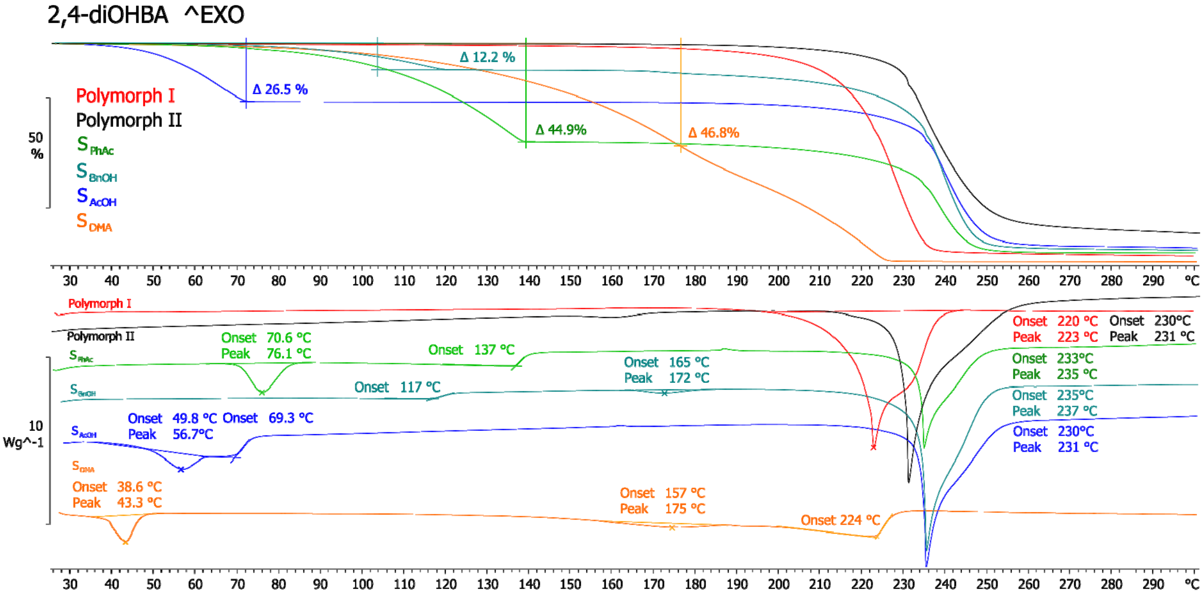

Figure S3. DSC-TG analysis of nonsolvated and undescribed solvated phases of 2,4-diOHBA.

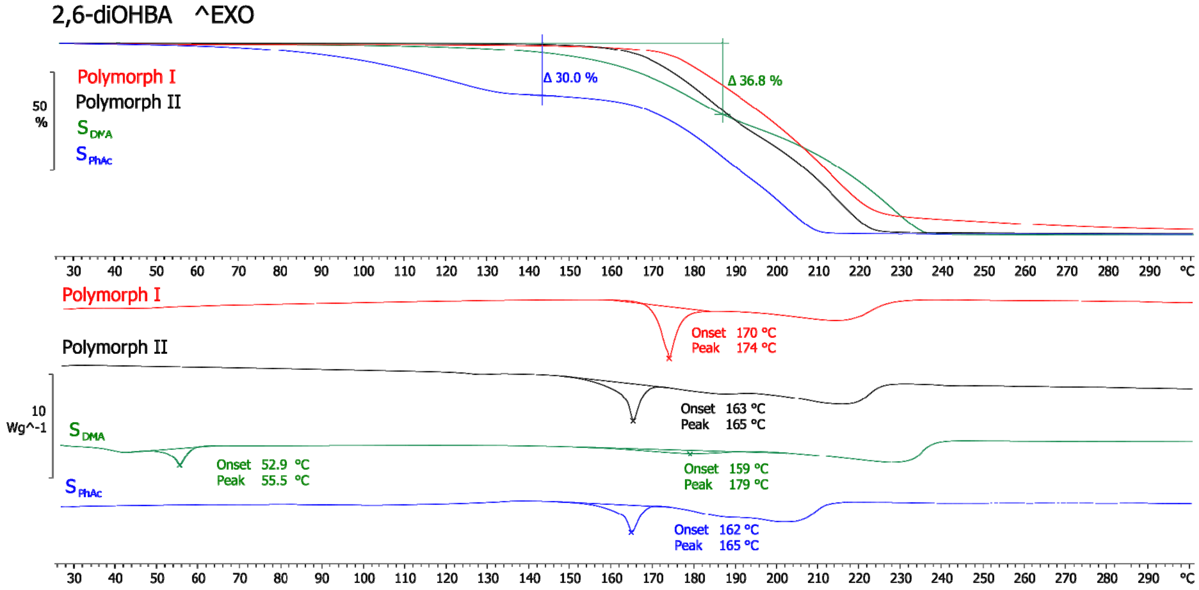

Figure S4. DSC-TG analysis of nonsolvated and undescribed solvated phases of 2,6-diOHBA.

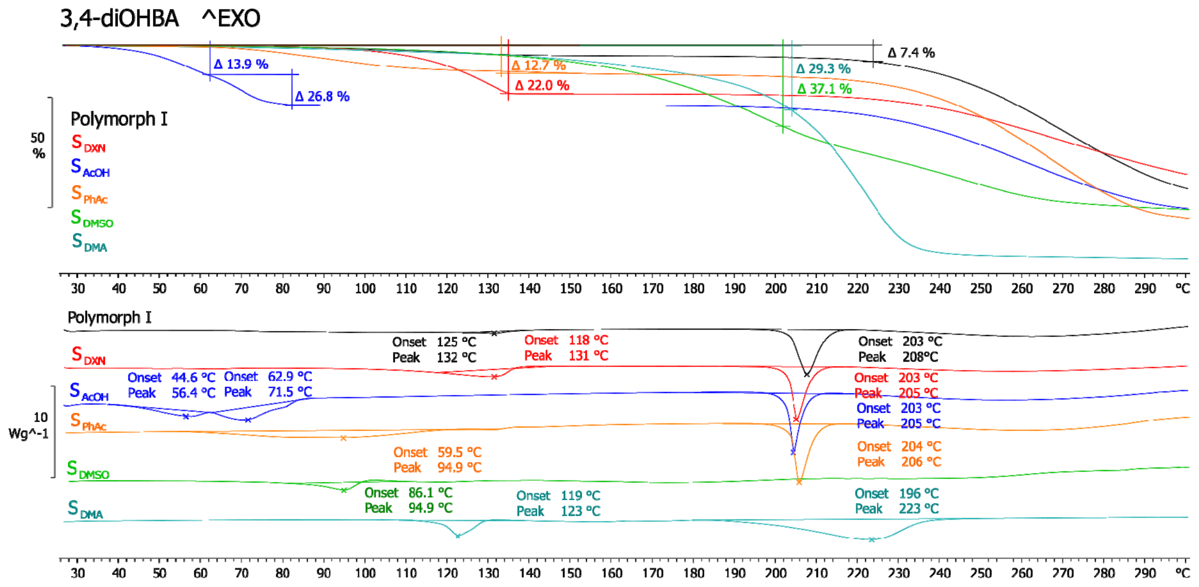

Figure S5. DSC-TG analysis of nonsolvated and undescribed solvated phases of 3,4-diOHBA.

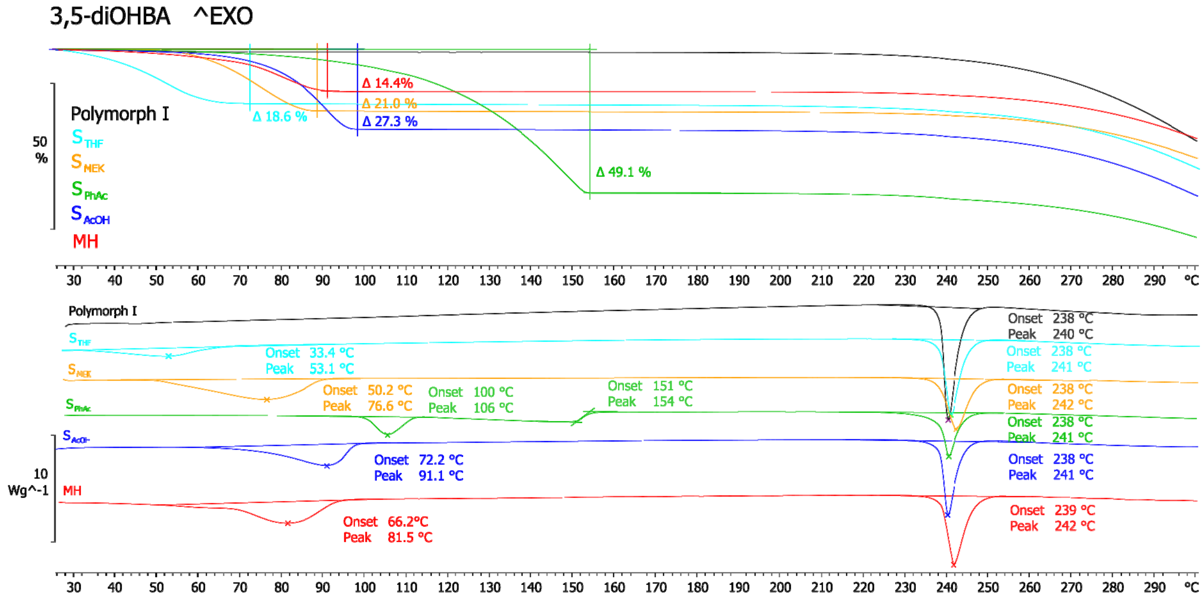

Figure S6. DSC-TG analysis of nonsolvated and undescribed solvated phases of 3,5-diOHBA.

Table S2. Crystal data and structure refinement details for newly characterized solvates.

Compound	3,4-diOHBA	3,5-diOHBA	3,5-diOHBA
Form	$S_{0.5\ DXN}$	$S_{0.5\ THF}$	S_{ACN}
Space group	P-1	P-1	P-1
a, Å	5.0168(1)	9.5853(5)	12.5934(4)
b, Å	9.5096(2)	9.9454(4)	12.6052(4)
c, Å	10.4088(3)	25.6021(5)	19.5070(9)
α,°	110.172(2)	79.195(2)	77.204(3)
β,°	102.084(2)	80.763(3)	87.380(3)
γ,°	95.278(2)	77.091(4)	67.527(3)
Z	2	8	4
Z′	1	4	2
Cell volume, Å^3	448.442(19)	2318.77(17)	2787.76(19)
Density$_{calculated}$, g·cm^{-3}	1.468	1.296	1.395
Temperature, K	293	293	150.0
Absorption coefficient μ, mm^{-1}	1.042	0.869	0.949
Packing index	70.9	65.0	70.2
θ range for data collection, °	4.6690–75.4540	4.7400–75.2710	0.9–75.0
Reflections collected/unique	8301/1780	25678/8699	46330/11599
GooF	1.070	1.053	1.041
Final R index (I > 2σ)	0.038	0.0728	0.0638
wR$_2$	0.119	0.2486	0.2109
Largest diff. peak and hole, Å^{-3}	0.138, −0.265	0.367, −0.332	0.380, −0.275

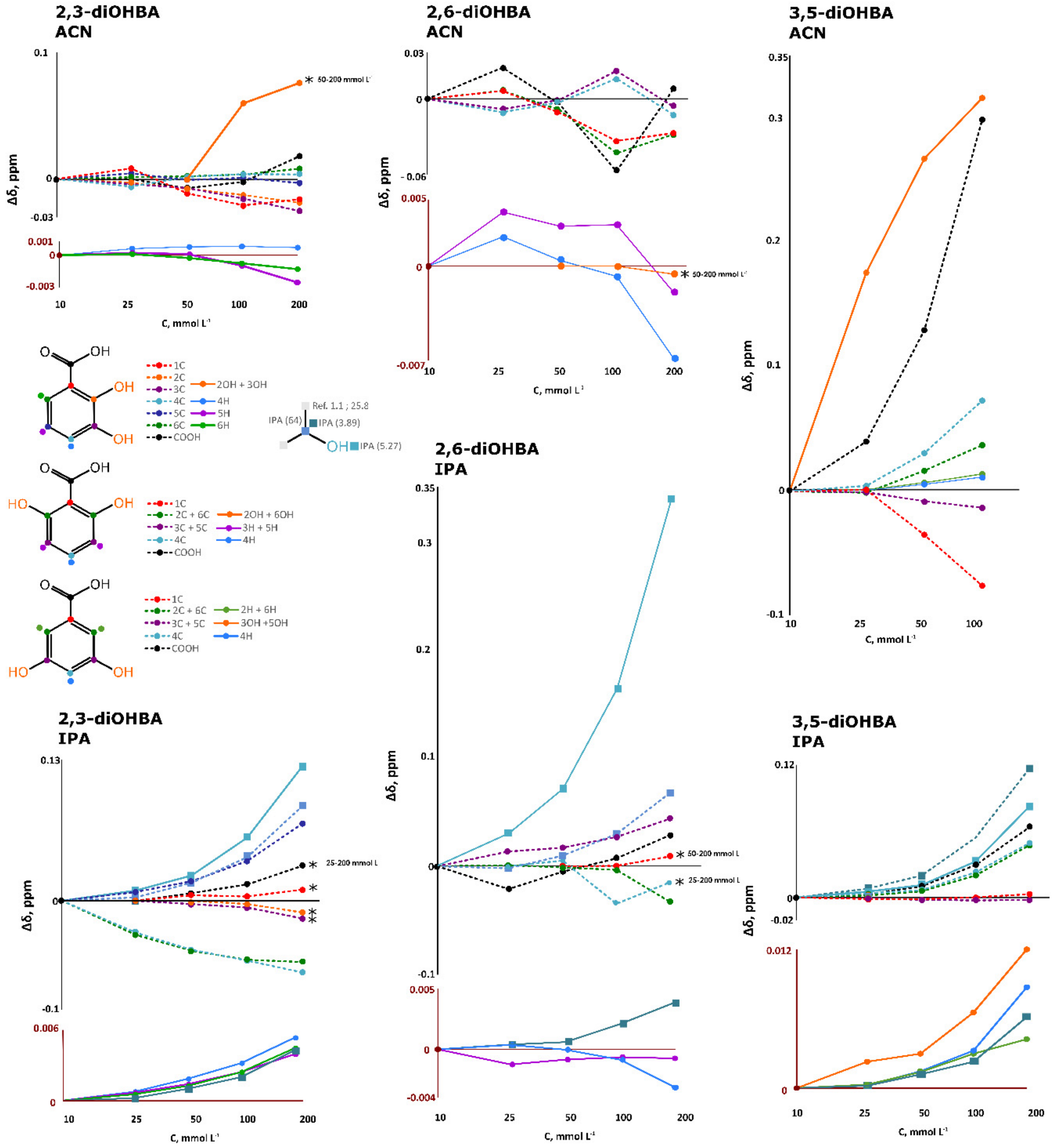

Figure S7. The ^{1}H and ^{13}C chemical shift dependence on concentration in the recorded NMR spectra of all 2,4-, 2,6- and 3,5-diOHBA solutions in acetonitrile and 2-propanol.

Logarithmic scale for the concentration axisis used. In all graphs two equal scale are used, see black and red axis. Points corresponding to the ^{13}C chemical shift changes are joined by dotted lines and those corresponding to ^{1}H are joined by solid lines, with both lines being guide for the eye. Color coding is used to assign each signal to a particular atom. Asterisks are used to indicate * – signal in not detected for the lowest concentration solutions.

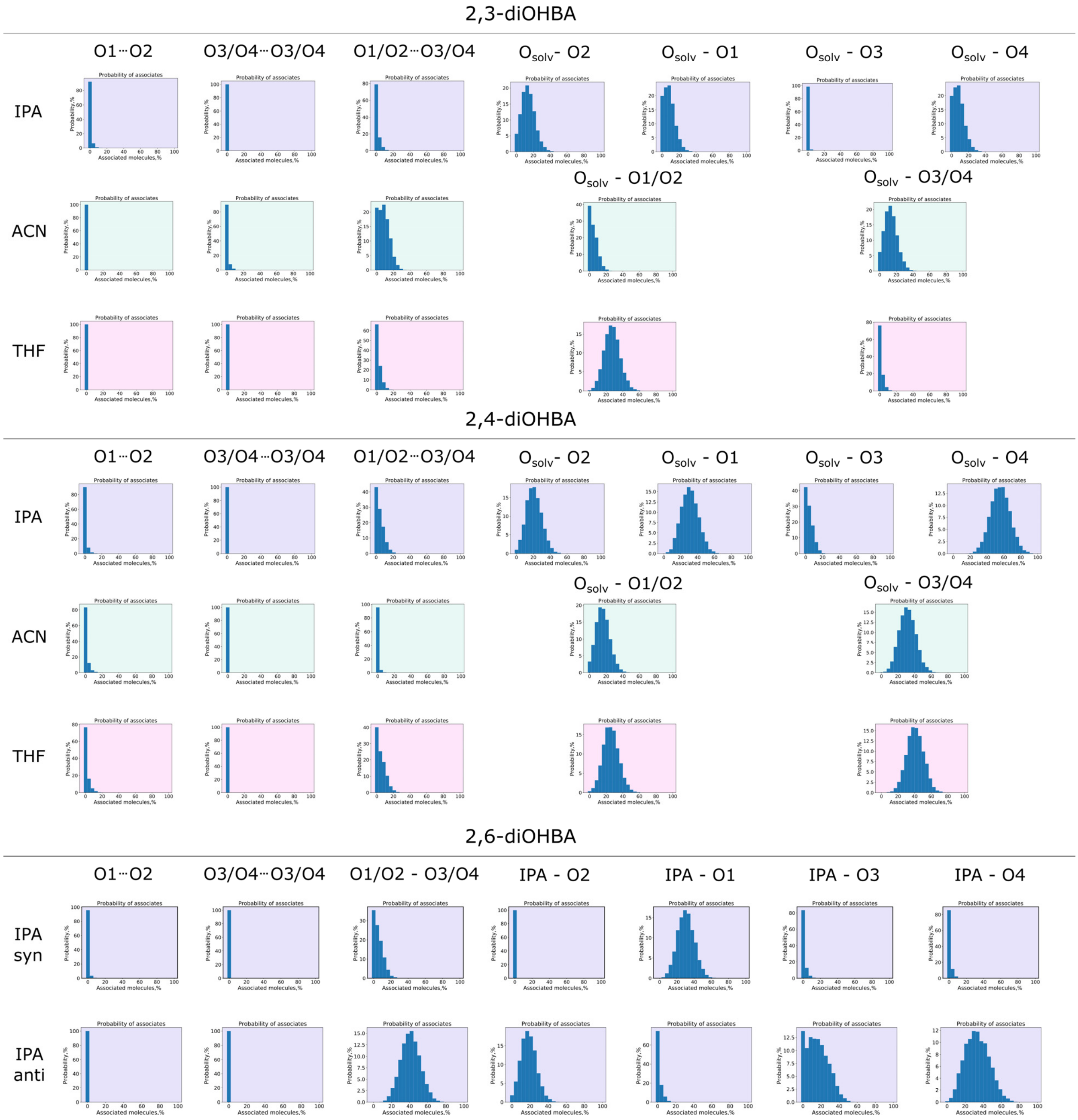

Figure S8. Probability for the selected Group A (2,3-diOHBA), Group B (2,4-diOHBA) and Group C (2,6-diOHBA, two conformations) compounds that the given relative amount of dOHBAs will be involved in a hydrogen bond between the specified O atoms of diOHBA and specified O atoms of diOHBA and hydroxyl group of 2-propanol (IPA), nitrile group of acetonitrile (ACN) and O atom of tetrahydrofuran (THF).

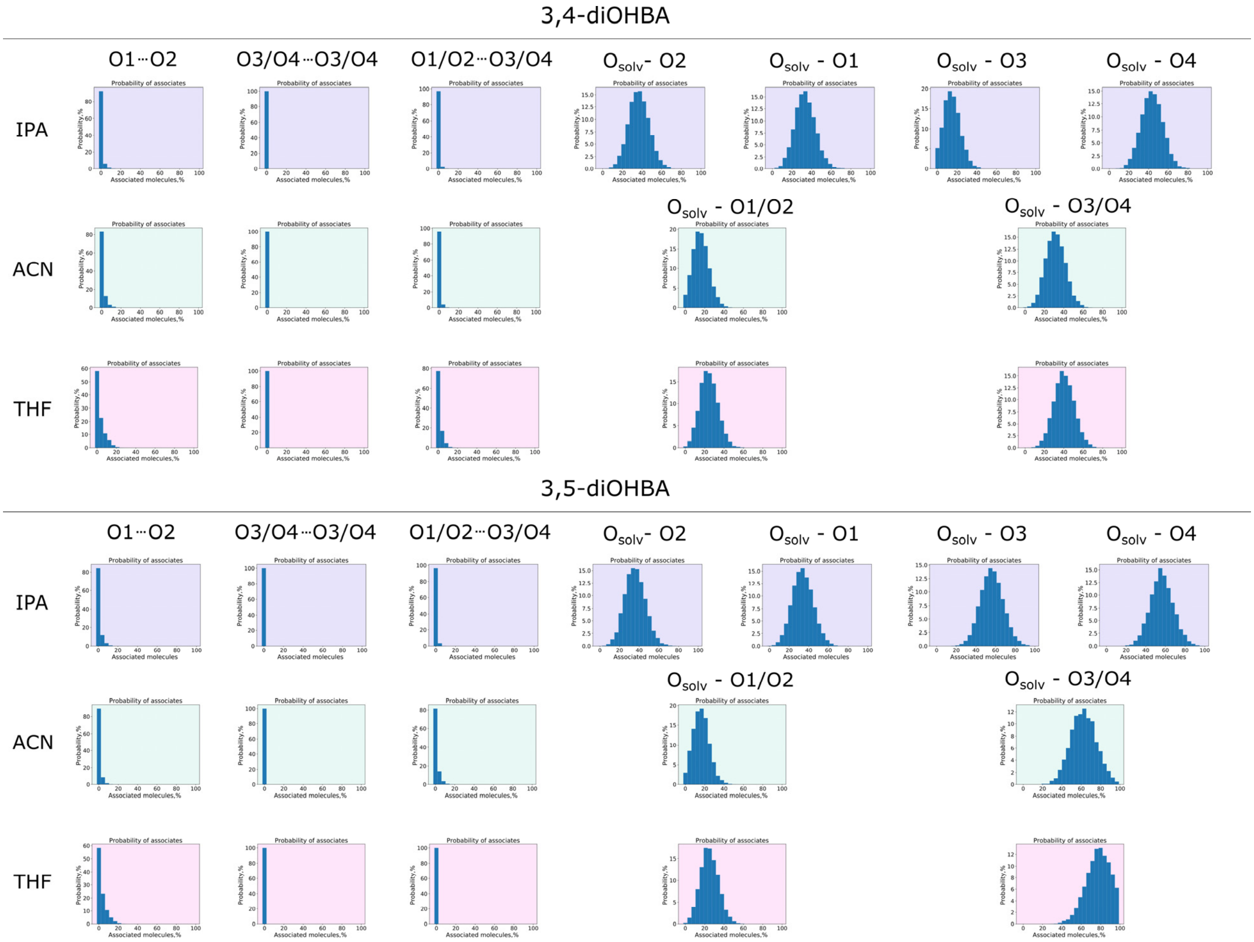

Figure S9. Probability for Group D (3,4-, 3,5-diOHBA) compounds that the given relative amount of dOHBAs will be involved in a hydrogen bond between the specified O atoms of diOHBA and specified O atoms of diOHBA and hydroxyl group of 2-propanol (IPA), nitrile group of acetonitrile (ACN) and O atom of tetrahydrofuran (THF).

Table S3. Summary of structural information on nonsolvated and selected solvated crystalline phases-crystallographic information, calculated packing index and lattice energy, obtained experimental melting enthalpies (nonsolvated phases) and the main hydrogen bond motifs.

Compound	Form	CSD identifier	Space group	Z, Z′	Packing index, %	Lattice energy, kJ·mol^{-1}	Enthalpy, J·g^{-1}	Intramolecular bond	$R^2_2(8)$	RHB motif
2,3-diOHBA	I	CACDAM[32]	$P\bar{1}$	4, 2	71.8	−112	−174	✓✓	✓	$R^{12}_{14}(34)$
	II	CACDAM01[38]	$P2_1/n$	4, 1	74.6	−110	−176	✓✓	✓	-
2,5-diOHBA	I	BESKAL01[33]	Pa	4, 2	71.8	−118	−205	✓	✓	$R^6_8(36)$
	II	BESKAL08[62]	$P2_1/n$	4, 1	73.7	−120	−194	✓	✓	$R^6_8(36)$
2,4-diOHBA	I	ZZZEEU08[34]	$P2_1/a$	4, 1	71.3	−114	*	✓	✓	$R^6_6(24)$, $R^6_8(36)$, $R^6_8(44)$
	II	ZZZEEU04[63]	$P2_1/n$	4, 1	74.1	−134	*	✓	✓	$R^6_8(36)$
	HH	QIVTUK[26]	$P\bar{1}$	2, 1	71.3	-	-	✓	✓	-
	MH	YUXGUAV[64]	$P\bar{1}$	2,1	70.6	-	-	✓	✓	-
2,6-diOHBA	II	LEZJAB[36]	$Pna2_1$	4, 1	72.2	−118	−115	✓✓	-	-
	I	LEZJAB01[35]	$P2_1/c$	4, 1	74.3	−123	−42	✓✓	✓	-
	MH	LEZJEF[35]	Pnma	8, 0.5	73.2	-	-	✓	-	-
3,4-diOHBA	I	WUYNUA[38]	$P\bar{1}$	6, 3	70.6	−139	−193	✓	✓	$R^6_6(34)$
	MH I	BIJDON03[26]	$P\bar{1}$	2, 2	73.6	-	-	✓	✓	-
	MH II	BIJDON04[38]	$P2_1/c$	4, 1	73.1	-	-	✓	✓	-
	S_{ACN}	EDUWUW[37]	$P2_1/c$	4, 1	71.9	-	-	✓	✓	$R^6_6(38)$
	$S_{0.5DXN}$	2077764	$P\bar{1}$	2, 1	70.9	-	-	✓	✓	-
3,5-diOHBA	I	WUYPOW01[38]	C 2/c	24, 3	71.0	−151	−266	-	✓	$R^6_6(40)$
	II	WUYPOW[38]	C 2/c	4, 0,5	71.6	−133	-	-	-	-
	HH	OKEMAT[39]	$P2_1$	2, 1	73.4	-	-	-	-	-

$S_{0,25THF}$MH	WUYPIQ[38]	Cc	4, 1	71.5	-	-	-	✓	$R^{6}_{6}(40)$
$S_{0.5THF}$	2077766	$P\bar{1}$	8, 4	65.0	-	-	-	✓	$R^{6}_{6}(40)$
S_{ACN}	2077765	$P\bar{1}$	4, 2	70.2	-		-	✓	$R^{6}_{6}(40)$
S_{DXN}	WUYPEM[38]	$P2_1/c$	4, 1	73.6	-		-	-	-

Gray—crystalline phase was not obtained during the crystal form screening.
Asterisk—the melting signal could not be distinguished from the decomposition and melting enthalpy could not be determined.

2,3-diOHBA

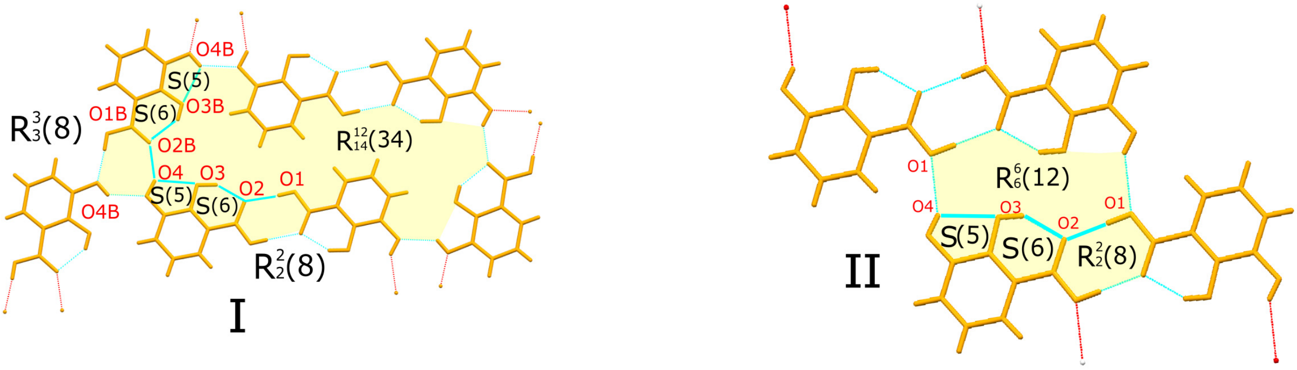

Figure S10. Hydrogen bond motifs in crystal structures of all known nonsolvated phases of 2,3-diOHBA.

Table S4. Hydrogen bond geometric parameters and motifs in crystal structures of all known nonsolvated phases of 2,3-diOHBA and corresponding hydrogen bond parameters.

Phase	Interaction	Distance, Å			D–H···A, °	Graph set	
		X–H	H···A	D···A			
I CACDAM	O1–H1···O2	0.84	1.85	2.685(3)	175	$R^2_2(8)$	R^{12}_{14} (34)
	O3–H2···O2	0.98	1.77	2.642(3)	147	S(6)	R^{12}_{14} (34)
	O3B–H2B···O2B	0.82	1.93	2.626(2)	143	S(6)	R^{12}_{14} (34)
	O4B–H3B···O3B	0.82	2.26	2.682(2)	113	S(6)	R^{12}_{14} (34)
	O4–H3···O3	0.88	2.24	2.699(3)	113	S(6)	R^{12}_{14} (34)
	O4–H3···O2B	0.88	2.05	2.809(2)	144	$R^3_3(8)$	R^{12}_{14} (34)
	O4B–H3B···O4	0.82	2.04	2.671(3)	133	$R^3_3(8)$	R^{12}_{14} (34)
	O1B H1B···O4B	0.83	1.81	2.645(2)	177	$R^3_3(8)$	R^{12}_{14} (34)
II CACDAM01	O1–H1···O2	0.89(3)	1.77(3)	2.661(3)	178(3)	$R^2_2(8)$	R^6_6 (12)
	O3–H2···O2	0.88(3)	1.81(3)	2.597(3)	148(3)	S(6)	R^6_6 (12)
	O4–H3···O3	0.79(3)	2.26(3)	2.689(3)	115(2)	S(6)	R^6_6 (12)
	O4–H3···O1	0.79(3)	2.32(3)	3.006(3)	145(3)	R^6_6 (12)	

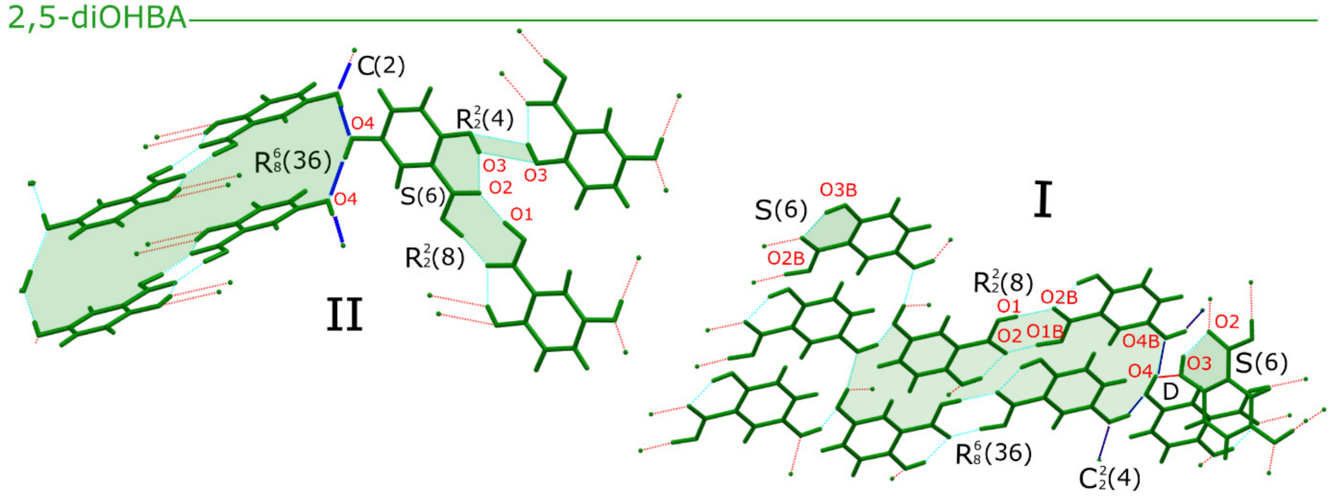

Figure S11. Hydrogen bond motifs in crystal structures of all known nonsolvated phases of 2,5-diOHBA.

Table S5. Hydrogen bond geometric parameters and motifs in crystal structures of all known nonsolvated phases of 2,5-diOHBA and corresponding hydrogen bond parameters.

Phase	Interaction	Distance, Å			D–H···A, °	Graph set	
		X–H	H···A	D···A			
II BESKAL08	O1–H1···O2	1.05(5)	1.68(5)	2.714(3)	171(5)	$R^2_2(8)$	R^6_8 (36)
	O3–H2···O2	0.85(4)	1.81(4)	2.577(4)	150(4)	S(6)	R^6_8 (36)
	O4–H5···O4	0.82(4)	1.95(4)	2.765(3)	176(2)	C(2)	R^6_8 (36)
	O3–H2···O3	0.85(4)	2.56(4)	3.109(4)	124(3)	$R^2_2(4)$	
I BESKAL01	O1–H1···O2B	0.86	1.81	2.666(2)	179	$R^2_2(8)$	R^6_8 (36)
	O1B–H1B···O2	1.14	1.54	2.672(2)	170	$R^2_2(8)$	R^6_8 (36)
	O3–H2···O2	0.90	1.87	2.630(2)	141	S(6)	R^6_8 (36)
	O3B–H2B···O2B	0.93	1.81	2.646(2)	148	S(6)	R^6_8 (36)
	O4–H5···O4B	1.02	1.86	2.769(2)	146	$C^2_2(4)$	R^6_8 (36)
	O4B–H5B···O4	0.96	1.96	2.723(2)	135	$C^2_2(4)$	R^6_8 (36)

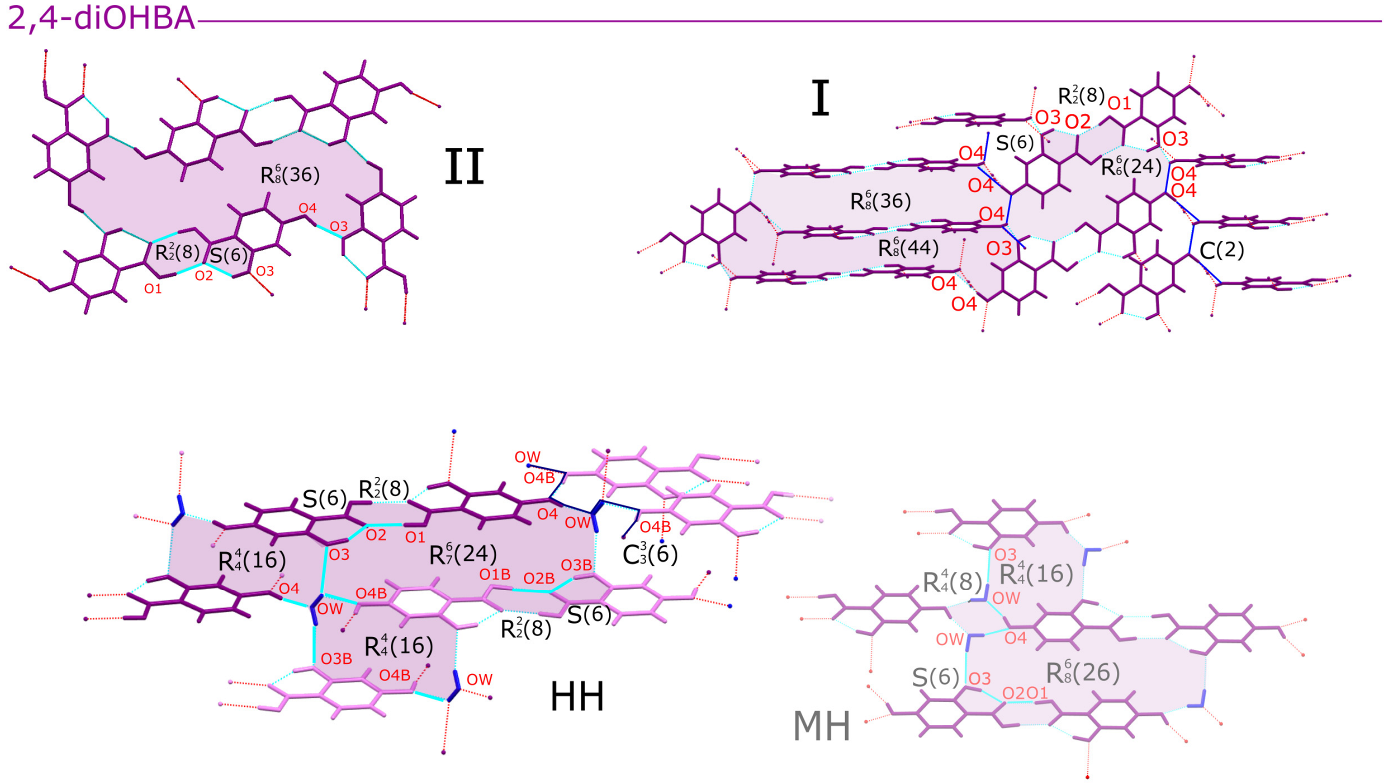

Figure S12. Hydrogen bond motifs in crystal structures of all known nonsolvated and hydrated phases of 2,5-diOHBA.

Table S6. Hydrogen bond geometric parameters and motifs in crystal structures of all known nonsolvated phases of 2,4-diOHBA and corresponding hydrogen bond parameters.

Phase	Interaction	Distance, Å			D–H···A, °	Graph set	
		X–H	H···A	D···A			
II ZZZEEU04	O1–H1···O2	0.90	1.76	2.658(2)	171	$R^{2}_{2}(8)$	$R^{6}_{8}(36)$
	O3–H2···O2	0.90	1.96	2.631(2)	130	S(6)	$R^{6}_{8}(36)$
	O4–H4···O3	0.87(3)	1.93(3)	2.782(2)	166(3)	D	$R^{6}_{8}3(36)$
I ZZZEEU08	O1–H1···O2	0.900(4)	1.749(3)	2.633(3)	166.5(4)	$R^{2}_{2}(8)$ $R^{6}_{6}(24)$	$R^{6}_{8}(36)$ $R^{6}_{8}(44)$
	O3–H2···O2	0.900(3)	1.881(3)	2.654(3)	142.9(3)	S(6) $R^{6}_{6}(24)$	$R^{6}_{8}(36)$ $R^{6}_{8}(44)$
	O4–H4···O3	0.900(4)	2.112(3)	2.827(3)	135.7(3)	D	$R^{6}_{8}(24)$ $R^{6}_{8}(44)$
	O4–H4···O4	0.950(3)	2.493(3)	3.302(3)	143.0(2)	C(2)	$R^{6}_{8}(36)$ $R^{6}_{6}(24)$

Table S7. Hydrogen bond geometric parameters and motifs in crystal structures of all known hydrated phases of 2,4-diOHBA and corresponding hydrogen bond parameters.

Phase	Interaction	Distance, Å			D–H···A, °	Graph set	
		X–H	H···A	D···A			
HH QIVTUK	O1 H1···O2	0.88(2)	1.79(2)	2.6774(16)	177(2)	$R^2_2(8)$	$R^6_7(24)$
	O1B H1B···O2B	0.87(3)	1.78(3)	2.6476(17)	175(3)	$R^2_2(8)$	$R^6_7(24)$
	O3 H2···O2	0.98(3)	1.72(3)	2.6082(17)	148(3)	S(6)	$R^6_7(24)$
	O3B H2B···O2B	0.88(2)	1.77(2)	2.5652(17)	150(2)	S(6)	$R^6_7(24)$
	O4 H4···OW	0.82(3)	1.80(3)	2.604(2)	168(2)	$R^4_4(16)$	$R^6_7(24)$
	OW HW1···O3	0.82(3)	2.40(3)	3.073(2)	141(3)	$R^4_4(16)$	$R^6_7(24)$
	OW–HW2···O3B	0.79(3)	2.05(3)	2.825(2)	165(3)	$R^4_4(16)$	$R^6_7(24)$
	OW–HW1···O4B	0.82(3)	2.29(4)	2.903(2)	132(3)	$R^4_4(16)$	$C^3_3(6)$
	O4B–H4B···O4	0.79(2)	1.94(2)	2.7266(18)	173.6(19)		$C^3_3(6)$
MH YUXGUV	O1–H1···O2	0.89(3)	1.78(2)	2.670(2)	175(2)	$R^2_2(8)$	$R^6_8(26)$
	O3–H2···O2	0.86(3)	1.81(2)	2.5937(19)	151(3)	S(6)	$R^6_8(26)$
	OW–HW1···O3	0.85(3)	2.07(3)	2.919(2)	173(3)	$R^4_4(16)$	$R^6_8(26)$
	OW–HW2···O4	0.78(3)	2.15(3)	2.825(2)	145(2)	$R^4_4(8)$	$R^6_8(26)$
	O4–H4···OW	0.83(2)	1.86(2)	2.678(2)	167(2)	$R^4_4(8)$	$R^4_4(16)$

Gray– crystalline phase was not obtained during the crystal form screening.

2,6-diOHBA

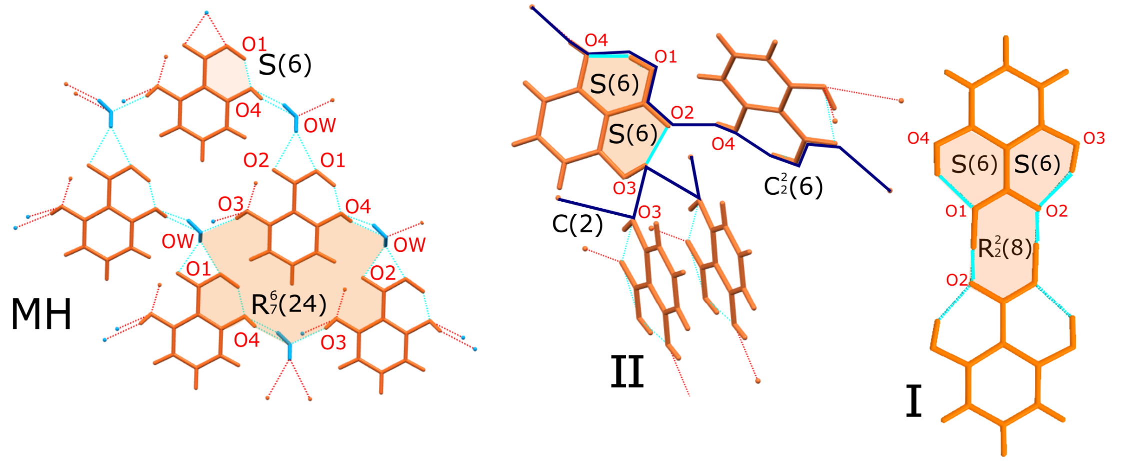

Figure S13. Hydrogen bond motifs in crystal structures of all known nonsolvated and hydrated phases of 2,6-diOHBA.

Table S8. Hydrogen bond geometric parameters and motifs in crystal structures of all known nonsolvated and hydrated phases of 2,6-diOHBA and corresponding hydrogen bond parameters.

Phase	Interaction	Distance, Å			D–H···A, °	Graph set	
		X–H	H···A	D···A			
MH LEZJEF	O1–H1···O4	1.14(14)	1.58(13)	2.540(3)	138(12)	S(6)	
	O1–H1···O1	1.14(14)	2.31(13)	2.938(3)	113(8)	D	
	OW–HW1···O4	0.86(11)	1.92(10)	2.705(4)	152(10)	$R^6_7(24)$	
	O4–H6···OW	0.71(6)	2.03(6)	2.705(4)	160(6)	$R^6_7(24)$	
	OW–HW2···O1	0.87(9)	2.33(9)	3.069(6)	143(6)	$R^6_7(24)$	
II LEZJAB	O1–H1···O4	0.89(4)	1.69(4)	2.522(3)	156(3)	S(6)	$C^2_2(6)$
	O3–H2···O2	0.94(3)	1.73(3)	2.596(3)	151(3)	S(6)	
	O3–H2···O3	0.94(3)	2.51(4)	2.928(4)	108(2)	C(2)	
	O4–H6···O2	0.76(3)	1.95(3)	2.683(3)	161(3)	$C^2_2(6)$	
I LEZJAB01	O1–H1···O2	1.33(5)	1.27(5)	2.603(3)	178(5)	$R^2_2(8)$	
	O3–H2···O2	0.93(4)	1.77(4)	2.603(3)	147(3)	S(6)	
	O4–H6···O1	1.00(4)	1.79(4)	2.584(3)	134(3)	S(6)	

3,4diOHBA

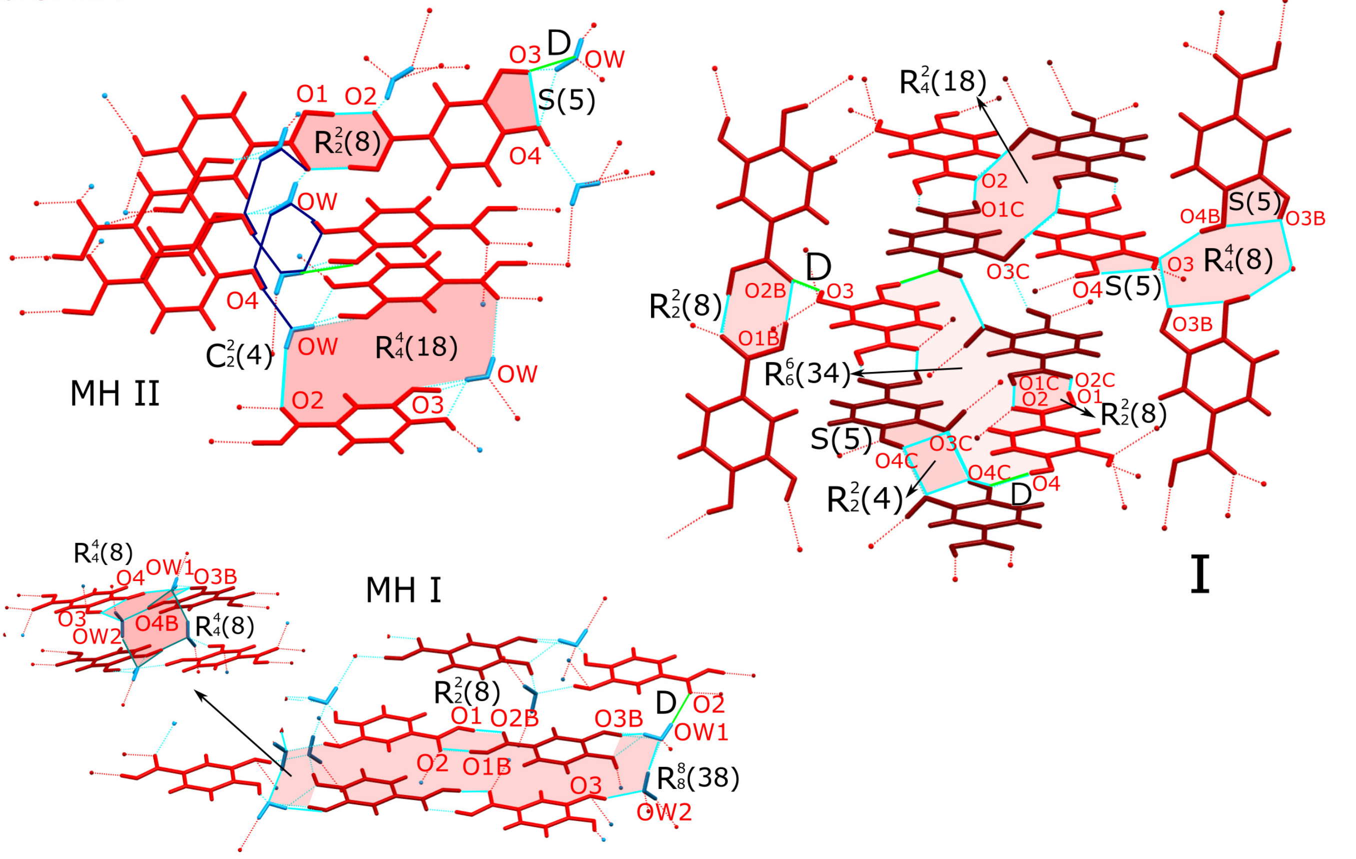

Figure S14. Hydrogen bond motifs in crystal structures of all known nonsolvated and hydrated phases of 3,4-diOHBA.

Table S9. Hydrogen bond geometric parameters and motifs in crystal structures of all known nonsolvated and hydrated phases of 3,4-diOHBA and corresponding hydrogen bond parameters.

Phase	Interaction	Distance, Å			D–H···A, °	Graph set	
		X–H	H···A	D···A			
MH II BIJDON04	O1–H1···O2	1.03	1.62	2.642(5)	172	$R^{2}_{2}(8)$	
	O3–H3···O4	0.99(10)	2.18(9)	2.634(4)	106(7)	S(5)	
	OW–HW1···O2	0.81	2.35	3.014(6)	140	$R^{4}_{4}(18)$	
	OW–HW2···O3	0.83	2.11	2.844(5)	148	D	
	OW–HW2···O4	0.83	2.42	3.036(6)	132	$C^{2}_{2}(4)$	
	O3–H3···OW	0.99(10)	1.91(10)	2.844(5)	157(8)	$R^{4}_{4}(18)$	
	O4–H4···OW	0.73	2.01	2.730(5)	172	$C^{2}_{2}(4)$	
MH I BIJDON03	O1–H1···O2B	0.874(2)	1.720(2)	2.592(2)	175.13(18)	$R^{2}_{2}(8)$	
	O1B–H1B···O2	0.918(2)	1.801(2)	2.717(2)	176.05(19)	$R^{2}_{2}(8)$	$R^{8}_{8}(38)$
	O3–H3···O4	0.911(2)	2.190(2)	2.655(2)	110.91(14)	S(5)	$R^{4}_{4}(8)$
	O3B–H3B···O4B	0.977(2)	2.137(2)	2.638(2)	110.12(14)	S(5)	$R^{8}_{8}(38)$
	OW1–HW1···O2	0.944(2)	2.053(2)	2.996(2)	176.99(18)	D	
	OW1–HW2···O3B	0.883(2)	2.230(2)	2.954(2)	139.08(19)		$R^{8}_{8}(38)$
	OW1–HW2···O4B	0.883(2)	2.386(2)	3.019(2)	128.82(18)	$R^{4}_{4}(8)$B	
	O3–H3···OW2	0.911(2)	2.058(2)	2.893(2)	151.75(16)	$R^{4}_{4}(8)$	$R^{8}_{8}(38)$
	O4–H4···OW1	1.047(2)	1.711(2)	2.733(3)	164.06(16)	$R^{4}_{4}(8)$	$R^{4}_{4}(8)$B
	O3B–H3B···OW1	0.977(2)	2.039(2)	2.954(2)	155.16(18)	$R^{4}_{4}(8)$	
	O4B–H4B···OW2	0.964(2)	1.765(2)	2.726(2)	174.21(16)	$R^{4}_{4}(8)$<br>$R^{4}_{4}(8)$B	$R^{4}_{4}(8)$B
	OW2–HW4···OW1	0.902(2)	2.080(2)	2.922(3)	154.92(19)	$R^{4}_{4}(8)$B	$R^{8}_{8}(38)$
	OW2–HW3···O3	1.030(2)	2.045(2)	3.012(3)	155.39(16)	$R^{4}_{4}(8)$	
I WUYNUA	O1–H1···O2C	1.03(6)	1.59(6)	2.606(5)	169(5)	$R^{2}_{2}(8)$	
	O1C–H1C···O2	0.90(5)	1.81(5)	2.694(5)	166(5)	$R^{2}_{2}(8)$	$R^{2}_{4}(18)$<br>$R^{6}_{6}(34)$
	O1B–H1B···O2B	0.94(7)	1.66(7)	2.595(5)	175(5)	$R^{2}_{2}(8)$	
	O3–H3···O4	0.82	2.19	2.648(4)	115	S(5)	$R^{4}_{4}(8)$
	O3B–H3B···O4B	0.82	2.11	2.591(5)	117	S(5)	
	O4C–H4C···O3C	0.77(5)	2.10(5)	2.657(6)	130(5)	S(5)	$R^{2}_{2}(4)$
	O4B–H4B···O3	0.82	1.99	2.769(4)	159	$R^{4}_{4}(8)$	
	O3B–H3B···O3	0.82	2.17	2.868(5)	143	$R^{4}_{4}(8)$	
	O3–H3···O2B	0.82	2.15	2.833(4)	141	D	
	O4C–H4C···O3C	0.77(5)	2.15(5)	2.730(6)	133(5)	$R^{2}_{2}(4)$	$R^{6}_{6}(34)$
	O3C–H3C···O2	0.96(8)	1.75(8)	2.702(6)	173(8)	$R^{2}_{4}(18)$	
	O4–H4···O4C	0.82	1.96	2.769(5)	168	D	$R^{6}_{6}(34)$

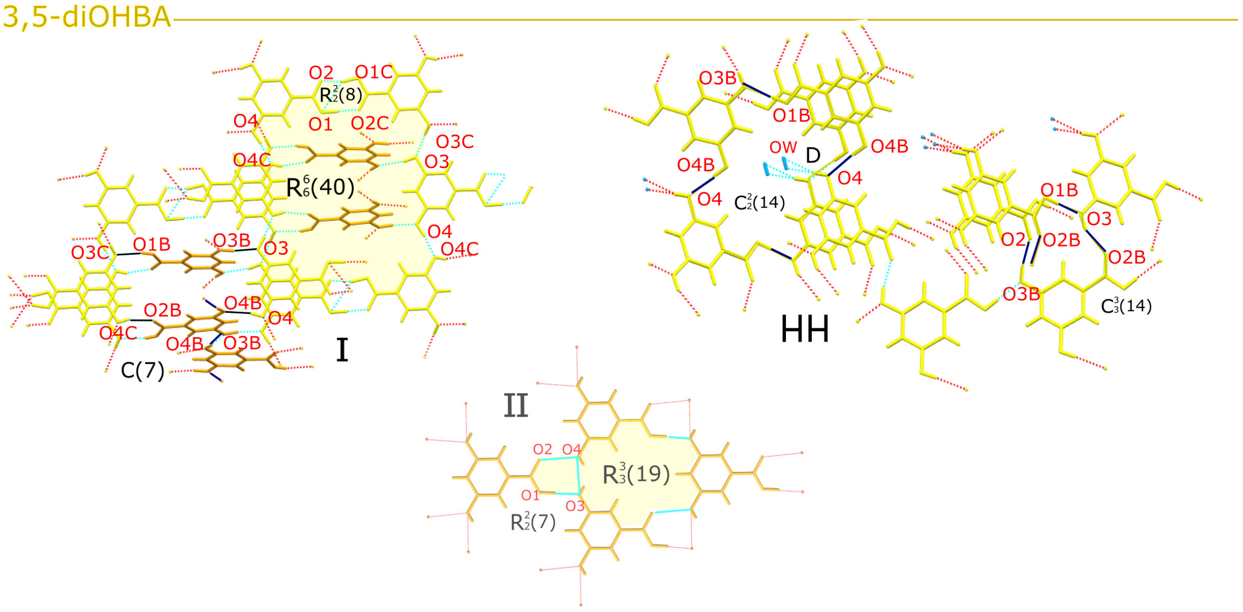

Figure S15. Hydrogen bond motifs in crystal structures of all known nonsolvated and hydrated phases of 3,5-diOHBA.

Table S10. Hydrogen bond geometric parameters and motifs in crystal structures of all known nonsolvated and hydrated phases of 3,5-diOHBA and corresponding hydrogen bond parameters.

Phase	Interaction	Distance, Å			D–H···A, °	Graph set	
		X–H	H···A	D···A			
I WUYPOW01	O1–H1···O2C	1.12(11)	1.54(11)	2.657(4)	179(19)	$R^{2}_{2}(8)$	$R^{6}_{6}(40)$
	O1C–H1C···O2	1.16(9)	1.51(9)	2.637(5)	163(10)	$R^{2}_{2}(8)$	$R^{6}_{6}(40)$
	O3–H3C···O3	0.80(4)	1.89(5)	2.689(6)	176(3)		$R^{6}_{6}(40)$
	O4–H5···O4C	0.98(11)	1.66(12)	2.636(6)	173(10)		$R^{6}_{6}(40)$
	O3B–H3B···O4B	0.81(7)	1.91(7)	2.719(5)	173(5)	C7	
	O3–H3···O3B	0.88(5)	1.91(5)	2.766(5)	163(5)	D	
	O4B–H5B···O4	0.94(5)	1.73(5)	2.671(4)	173(6)	D	
	O1B–H1B···3C	0.77(7)	1.90(7)	2.648(5)	163(6)	D	
	O4C–H5C···2B	0.79(7)	1.92(7)	2.681(5)	161(8)	D	
HH OKEMAT	O4B–H5B···O4	0.84	1.97	2.809(11)	178	$C^{4}_{4}(26)$	
	O1B–H1···O3B	0.84	1.84	2.663(9)	165	$C^{4}_{4}(26)$	
	O1–H1···O3	0.84	1.84	2.664(9)	167	$C^{4}_{4}(26)$	$C^{3}_{3}(14)$
	O4–H5···OW1	0.84	1.92	2.716(15)	158	D	
	O4–H5···OW2	0.84	1.93	2.762	170'	D	
	O3B–H3B···O2	0.84(6)	1.84(9)	2.663(8)	168(16)	$C^{3}_{3}(14)$	
	O3–H3···O2B	0.84(4)	1.88(6)	2.661(8)	155(10)	$C^{3}_{3}(14)$	
II WUYPOW	O1–H1···O4	1.04(6)	1.68(6)	2.7131(18)	177(6)	$R^{3}_{3}(7)$	$R^{3}_{3}(19)$
	O4–H5···O4	0.63(5)	2.11(6)	2.640(2)	143(5)	$R^{3}_{3}(7)$	$R^{3}_{3}(19)$

Gray– crystalline phase was not obtained during the crystal form screening.

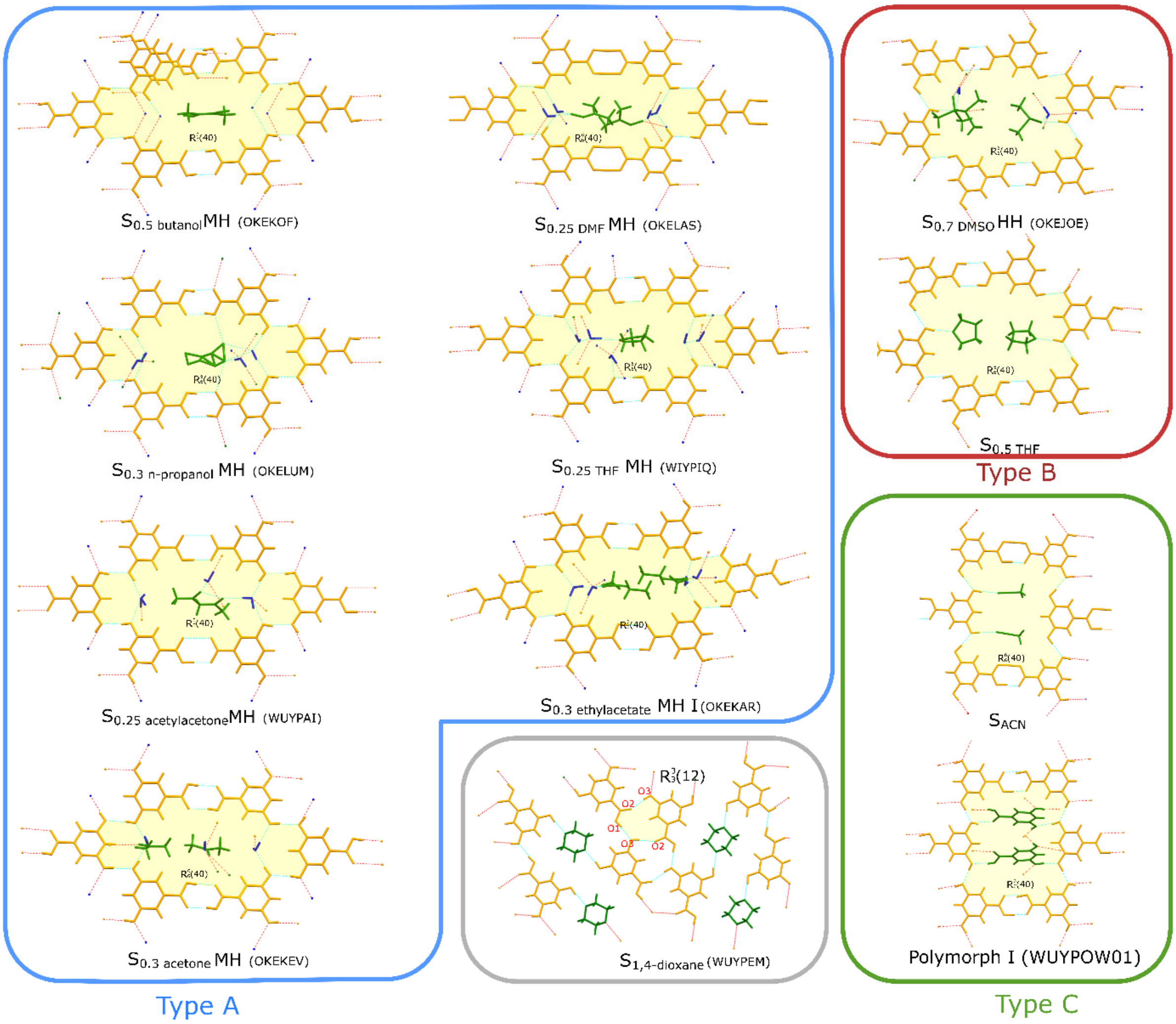

Figure S16. The identified types of 3,5-diOHBA solvated forms based on the RHB motif in their structure and hydrogen bond motifs in crystal structure of 3,5-diOHBA 1,4-dioxane solvate.

Type A solvates–RHB motif has elongated shape and water molecules are mandatory. Type B solvates–RHB motif is skewed, and water molecules are not mandatory. Type C solvate–RHB motif is identical to the RHB motif in 3,5-diOHBA polymorph I.

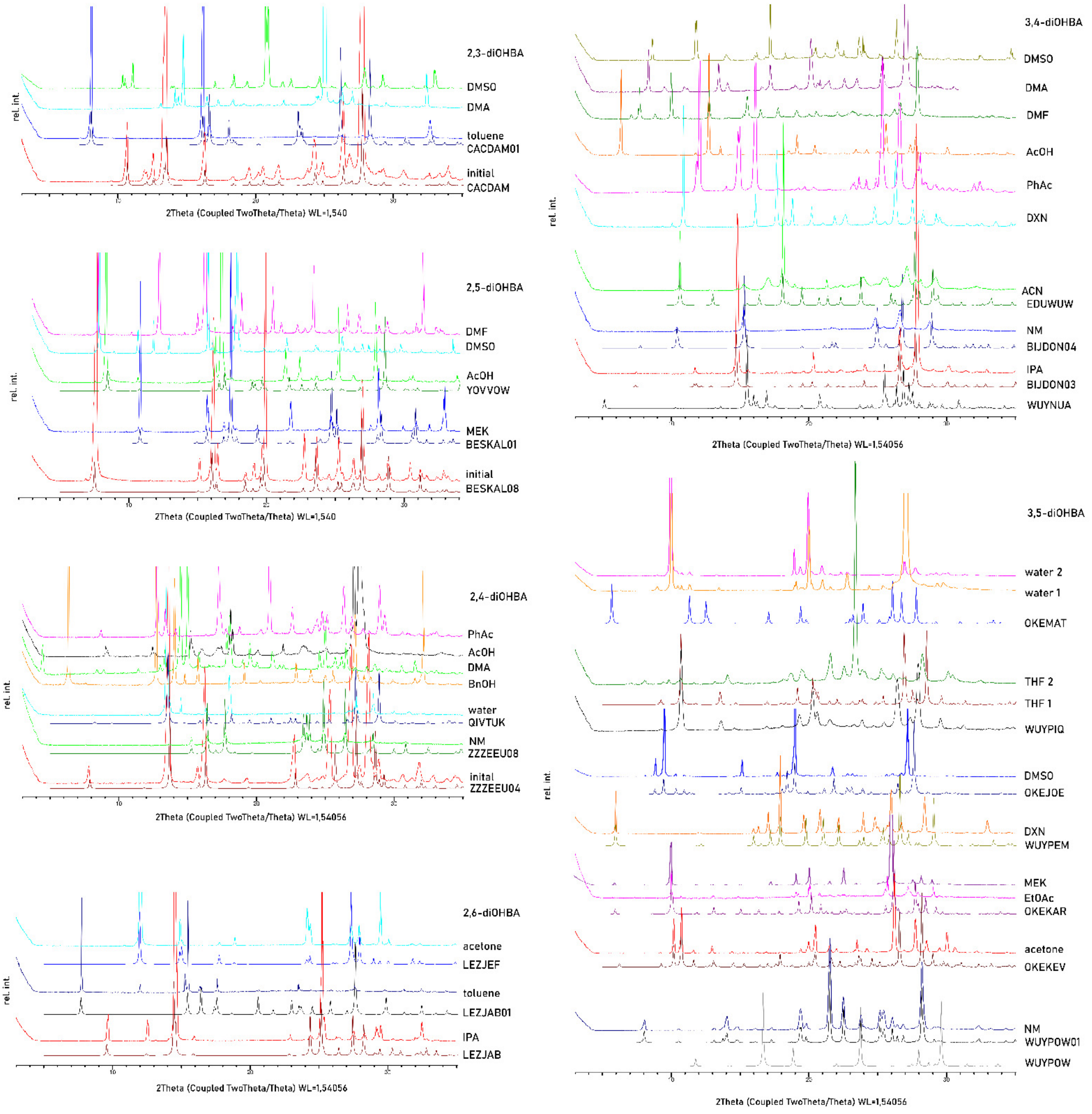

Figure S17. Phase identification of selected obtained products using PXRD patterns simulated from the crystal structures deposited in the CSD.
